# Leaders' Networking Behaviours in a Time of Crisis: A Qualitative Study on the Frontline against COVID‐19

**DOI:** 10.1111/joms.12884

**Published:** 2022-12-12

**Authors:** Stefano Tasselli, Alessandro Sancino

**Affiliations:** ^1^ University of Exeter; ^2^ Erasmus University; ^3^ Università degli Studi di Milano Bicocca; ^4^ Open University

**Keywords:** networking, social networks, microfoundations, leadership, behaviours, nomothetic, idiographic, organizational crisis, COVID‐19, qualitative research, cluster analysis

## Abstract

What do leaders do when they interact with followers and stakeholders in a time of crisis? What networking behaviours do leaders manifest in such a context of emergency? We answer these questions through qualitative research and cluster analysis conducted on a sample of leaders involved in community management in the most affected region in northern Italy during the three key phases of the COVID‐19 pandemic. Our findings span a period of 18‐months and show that leaders display a behavioural repertoire that includes six networking actions. Grouped together, these actions identify three clusters of leaders: Churners, who engage mainly in network generation and network termination; Divergent leaders, who manifest high levels of network conflict and re‐construal; and Sense‐makers, who are high in network deepening and teleology. Our research contributes to unveil the idiographic micro‐foundations of networking behaviour during organizational jolts.

## INTRODUCTION

Leadership research has long wrestled with the question of whether and how the relationships that leaders experience with followers and stakeholders influence organizational functioning (e.g., Balkundi and Harrison, 2006). From a network perspective, leadership is a relational process involving actors across multiple levels of analysis, from dyads and groups to organizations and societies (e.g., Brass and Krackhardt, 1999; Zohar and Tenne‐Gazit, 2008). From this approach, leadership resides ‘not in the attributes of individuals’, but in the ‘relationships connecting individuals’ within and across the social space of the organization (Balkundi and Kilduff, 2006, p. 420).

Extensive research provides evidence that interpersonal ties are instrumental to the leadership role (for a recent review, see Carter et al., 2015). They yield access to knowledge, social support and a variety of important resources (e.g., Burt et al., 2013). The occupation of specific positions in different social networks, such as advice or friendship, might explain the extent to which individuals occupy leadership roles (e.g., Parker and Welch, 2013), are perceived as charismatic by followers (Balkundi et al., 2011), and develop their reputation as leaders among different organizational constituencies (Mehra et al., 2006). Moreover, the relational benefits of a leader's network move beyond the leader: the leader's position in a team's network influences performance within (Balkundi et al., 2009) and external to the team (e.g., Morgeson et al., 2010); and the position occupied by a formal leader in the larger organizational network affects the followers' potential to be themselves influential (Sparrowe and Liden, 2005).

However, previous research on leadership networks restricted the understanding of leaders' influence on followers and organizations to the analysis of either formal or informal authority structures (e.g., Oh et al., 2004; Sparrowe and Liden, 1997), privileging a view of leaders' actions as ‘heavily embedded in social relations’ (Granovetter, 1985, p. 482). Consequently, much of existing research falls short in describing what leaders *do* when they engage in networking, i.e., the processes underlying leaders' *behaviours* in social interactions.

We counterbalance the structural view of leadership with a micro‐foundational lens that puts leaders' behaviour and decision making back into focus. We give emphasis to the ways leaders behave and take action in social networks, such that the leader ‘derives its meaning and its potential for action from relations of multiple kinds’ with followers and stakeholders (Shipilov et al., 2014, p. 449). And, relatedly, we suggest that leaders' networks are shown to emerge from the patterns through which ‘localized actions, relationships, and identities cohere into higher‐level network structures’ (Tasselli et al., 2015, p. 1378). The opportunity for this micro‐foundational analysis of the emergence of networking behaviour derives in this empirical research from the ‘system of emergent complexity’ (Kilduff et al., 2008, p. 85) triggered by the management of COVID‐19, which represents a unique laboratory to observe leaders' relational behaviour (e.g., Muzio and Doh, 2020; Uhl‐Bien, 2021). The pandemic crisis, in this sense, is not just a setting or a contingency, but may be epistemologically considered as an ‘epiphenomenon of life itself’ (Granovetter, 1985, p. 482), an opportunity to shed light on what leaders *do* when they engage in interactions with others that are not prescribed by existing structural arrangements.

We build on this insight to examine, through qualitative inquiry and cluster analysis, the behaviours of leaders involved in the response to COVID‐19 in four of the most affected provinces in the Italian Lombardy region. Our study consisted of three phases, with interviews with leaders conducted during the peak of the first (February – April 2020) and of the second wave of the viral infection (November – December 2020) and during a third phase (June – July 2021) in which, following the vaccination campaign, the crisis seemed to be successfully contained. Our emphasis is on the leaders' networking behaviours during this time of emergency.

How did leaders behave in their interactions with followers and other stakeholders during the COVID‐19 crisis? Following the organizational disruption in the midst of the pandemic, did leaders exhibit consistent patterns of relational actions that can help us detect and understand their network‐related behaviours? These are the questions leading our research. Evidence of repeated behavioural patterns (or, using the label suggested by Tasselli et al. (2015), ‘behavioural signatures’) emerged from the interviews that we conducted and guided our analysis. In the qualitative study, we found that leaders tended to focus on six leading actions, which describe ‘what leaders do’ when they engage in networking with others (e.g., Vissa, 2012). These actions represent a broad repertoire of behavioural categories that leaders manifest in their interpersonal interactions in the context of crisis. They identify behavioural traces of ego's networking that transcend structural roles or positions. They include network generation and termination (both belonging to a structural domain of action), network conflict and deepening (belonging to a network utilization domain of action), and network teleology and re‐construal (belonging to a network interpretation domain of action) (for representative categories and quotes, see Table II). We discuss the theoretical foundations of these actions, as well as the links of each of them with concepts developed by previous literature, in Table III. Through cluster analysis, we then found that the networking behaviours of the 42 leaders included in our sample could be grouped and categorized in three clusters, which we labelled Churners, Divergents and Sense‐makers (see Table IV).

We make one main contribution to theory and research on networks and leadership: we contribute to unveil the micro‐foundations of networking behaviour during organizational jolts. We have little knowledge of the processes by which leadership involves relational actions. We have even less knowledge on people's networking behaviours in a time of crisis, i.e., when organizational structures and routines are shaken up (Tasselli, 2019). Despite the growing attention of social network scholarship to networking (e.g., Halevy et al., 2019), lay‐theories (e.g., Kuwabara et al., 2018) and behavioural processes and strategies (e.g., Obstfeld et al., 2014; Quintane and Carnabuci, 2016), none of this work investigated so far how leaders engage in networking behaviour in a time of crisis. The setting of this study is particularly suitable to answer these questions, because the emergency associated with the pandemic changed the structural network routines underlying leaders' behaviours, opening the door to the investigation of questions related to the psychology of conflict, meaning, and construal in network behaviour in a context of emergent complexity.

Specifically, we answer these questions following an idiographic approach that allows us to capture the contingent and even subjective meaning of the underlying patterns of leaders' relationships and behaviours. Compared to nomothetic approaches, which tend to treat social phenomena as categorically and prototypically objective (e.g., Windelband, 1998), idiographic views help researchers to focus on what Guicciardini (1530/1972) called *particulare*, i.e., on the Kantian awareness that social reality is framed and reconstructed through context‐specific and individual sets of events and behaviours that require in‐depth and, in some cases, individual‐based investigation (Münsterberg, 1899). With respect to the analysis of leadership networks (e.g., Carter et al., 2015), our idiographic lens bridges research that looked at leadership *in* networks (i.e., at social networks as antecedents of leadership emergence; e.g., Lau and Liden, 2008; Oh et al., 2004), as discussed in our presentation of ‘churning’ leaders; and research that looked at leadership *as* networks (i.e., at how individuals perceive the leadership relationships in their social contexts; e.g., Graen and Uhl‐Bien, 1995), as discussed in our presentation of ‘sense‐making’ leaders. By escaping structural heuristics and pursuing rich, context‐specific analysis, we call for a paradigm shift on research in the networking behaviours not only of leaders but, more in general, of organizational members. Idiographic approaches, in our view, can compensate the limits inherent in structural analysis and help understand people's social behaviours as ‘embedded in concrete, ongoing systems of social relations’ (Granovetter, 1985, p. 487).

### An Idiographic Approach to Leaders' Networking Behaviours in a Time of Crisis

Social network approaches to leadership tend to portray leaders' behaviour as embedded in structures that regulate decision‐making and leadership functioning (e.g., Balkundi and Kilduff, 2006). The assumption is that leaders' actions are implicitly captured by their structural positions in the network. This almost exclusive structural focus on *networks*, i.e., on sets of roles and positions that are assumed to influence leaders' outcomes in organizations, has not been paralleled for long time by an adequate development of the study of *networking*, i.e., the study of how leaders behave in their social worlds.

This dualism between networks (structures) and networking (behaviours) has fuelled a longstanding debate on the agency of leaders in network contexts. Already decades ago, Dennis Wrong (1961) criticized the ‘over‐socialized conception’ of individual actors in structural network research, following Parsons's (1937) emphasis on structural orders – and on fix and hierarchically imposed roles – as a way to give sense to otherwise fleeting relationships. However, when detached from situated action and behaviour, leaders' networks risk becoming super‐structural, and thus extraneous, to the leaders themselves who forge and maintain interpersonal interactions with others. Leaders' behaviours and even their local relationships are ultimately ‘epiphenomenal in comparison with enduring structures of normative role prescriptions’ (Granovetter, 1985, p. 486). Emphasis on structure tends to neglect the qualitative understanding of networking, obscuring the importance of the actions and behaviours exerted by leaders in their localized and subjective relationships with stakeholders.^[^ ^1^ ^]^

Aiming at counter‐balancing the over‐reliance of previous research on structure as a network correlate of leadership, a major crisis, such as the pandemic, might become the opportunity for ‘altering the organizational and occupation structure of work’ (Barley, 1986, p. 78) and observing (relatively) unconstrained behaviours manifested by the leaders. Citing classic work on the balance between behaviour and structure by Mead (1932, p. 71), ‘What drives the awakening of consciousness from one level to the next is the “awakening of delayed and conflicting responses” to problematic situations in one's various environments’ (in Emirbayer and Mische, 1998, p. 969). Social facts, including the ways leaders behave in the practice of leading, are ‘ecologically embedded’ within specific contexts of time and space (Emirbayer and Goodwin, 1994, p. 1416), to a point that behaviours can be even defined as ‘structures in the process’ (Abbott, 1992, p. 14), or stratified models of action (see Giddens, 1979). A time of crisis can be seen as an epistemological window for sense‐making (e.g., Christianson and Barton, 2021), in which functionalist notions such as ‘role’, ‘activity’ and ‘interaction’, which have been used by many network scholars in a structural fashion (e.g., White et al., 1976), might be re‐conceptualized as elements of leaders' ‘actions in interactions’ (Tasselli and Kilduff, 2021).

This consideration is particularly relevant in the ongoing scholarly discussion on the role of behavioural networking (e.g., Halevy et al., 2019). There is substantial agreement in the literature that behaviours generally escape the normative boundaries of structure, such that they can be enacted ‘regardless of the network structure in which one is embedded’ (Grosser et al., 2019, p. 115; see also Obstfeld et al., 2014); they are typically construed as ‘domain‐specific’ systems of action (Kuwabara et al., 2020, p. 2) and so grounded in the contingent social reality in which they are manifested; and they tend to explain individuals' localized actions and outcomes above and beyond structural contingencies (e.g., Obstfeld, 2005). The debate is progressively moving away from the view of behaviours as ‘general orientations’ (Grosser et al., 2019, p. 121), addressing the localized questions of (i) whether behaviours are idiosyncratic and unique for specific groups of individuals; and (ii) whether they are located in a specific situational and ecological context to explain the ways a leader behaves when in a position.

Empirically, this theoretical shift requires a parallel change from the use of nomothetic methods (i.e., those that aim to identify specific variables that can be measured and tested across individuals irrespective of the situation) to idiographic methods (i.e., those that identify complex patterns of behaviour *within the person* that emerge in specific *experiences or situations*). In the nomothetic approach, traditionally used by research analysing the relationships between personality traits (e.g., Mehra et al., 2001), behavioural orientations (e.g., Obstfeld, 2005) and networking, each individual is measured in respect to one or multiple variables in time: the individual is ‘atomized’ in the measurement of specific dimensions, or scales (Allport, 1937). On the contrary, idiographic approaches yield ‘within‐person’ patterns: whether certain cognitions, strategies, or behaviours are ‘yoked together in time for a particular individual’ (Conner et al., 2009, p. 294). This is an account of individuality consisting of situation‐based networking behaviours that follow a so called ‘if‐then’ logic: *Certain* individuals manifest and adjust *certain* behaviours according to *certain* [social] situations, and they do so consistently and idiosyncratically (Mischel and Shoda, 1995). For example, if a certain situation (such as the COVID‐19 emergency) occurs, then certain individuals (or clusters of individuals) will behave in a certain way.

To operationalize this argument in our qualitative analysis, leaders' networking behaviours (a) are relevant for certain leaders in certain social situations, (b) consist of multiple and consistent sets of networking actions that (c) emerge and result in the same behavioural pattern for the same kinds of leaders in the given situation. For example, the networking behaviours that characterizes Sense‐makers (a) are relevant for leader Chris (fictional name; respondent 13 in Appendix 1) – but not for leader Joe (fictional name; respondent 38 in Appendix 1, who is grouped in a different cluster) – in the context of crisis triggered by COVID‐19; (b) are manifested through relational actions that include deepening and teleology (see Table III for a description of networking actions); and (c) are consistent, in the same emergent situation, with the behaviours of Alex and Anna (fictional names; respondents 35 and 42 in Appendix 1), who are indeed classified in Chris' same behavioural cluster.

Overall, we aim at embracing the idiosyncratic complexity of leaders' behaviours following an approach oriented at deconstructing the nexus between action and behaviour through an account of individuality that consists of situation‐based behaviours. Specifically, we contextualize and extend the behavioural study of networking to an empirical context characterized by high uncertainty and unpredictability, casting novel theoretical and managerial insights whose implications go beyond the COVID‐19 crisis.

## METHODS

### Research Setting

We conducted a three‐phase interpretive qualitative study on the networking of leaders facing COVID‐19 in four of the most affected provinces (Milano, Bergamo, Brescia, Monza‐Brianza) in the most affected Italian region (Lombardy). We interviewed the same sample of leaders three times, during the first two waves of the pandemic (winter and spring 2020 and autumn 2020) and during a third phase (spring and summer 2021) in which the success of the vaccination campaign was paralleled by a temporary decrease of the emergency. In the first wave of the viral infection (winter and spring 2020), Lombardy was the first Western region facing the health, social and organizational consequences of COVID‐19, which put under extreme pressure not only the health care system, but also the management of most organizations and communities. Italy has been one of the first countries entering total lockdown in early 2020, i.e., restricting possibilities of movement and activity for its citizens. In autumn 2020, during the second wave of the viral infection, Lombardy was still heavily affected by COVID‐19. We restricted the analysis to specific time intervals in these three phases of the pandemic, given their relevance for the management of the crisis: during the first wave, we collected data in the two months ranging from 21 February 2020, starting date of the outbreak in Italy, to the week of 21 April 2020, when the national government announced the plan of re‐opening after the total lockdown, thus starting a new phase after the first peak of the emergency. This first phase represented the first peak of the pandemic. During the second wave, we collected data related to the five‐week period between 15 November 2020, when the Region was in total lockdown due to the rising number of infections, and 21 December 2020, right after the Region relaxed the lockdown because of a reduction in the number of cases. The third data collection respected the same 6–7 month time interval between the first two phases but, different from the first phases, was conducted in a moment of non‐emergency: it started indeed at the beginning of June and ended in late July 2021. Although conceptually different from the first two phases (which had a narrow focus on the leaders' reaction to the peaks of the emergency), this third follow‐up phase mainly served to provide overall validity for the findings and to test patterns of stability or variability in behaviours in a post‐emergency moment of the pandemic (See Table I for an overview of the phases and chronological distribution of the interviews; see Appendix 1 for a detailed description of the respondents). Our setting was ideal in answering our research questions, providing access to unique data on the leaders' management of the crisis in the area that represented for many weeks both the Western epicentre and the organizational archetype of the emergency.

**Table I:** Overview of the interview data for chronological phase

Phase	Number of interviews	Gender of the leaders	Formal/informal leaders	City size (number of inhabitants)	Geographical area
Phase 1 (February – April 2020)	42	F = 36%; M = 64%	81% formal leaders; 19% informal leaders	36% < 15.000; 43% < 50.000; 11% < 100.000; 10% > 100.000.	62% Milano area; 12% Bergamo area; 10% Brescia area; 16% Monza and Brianza area.
Phase 2 (November – December 2020)	35	F = 34%; M = 66%	80% formal leaders; 20% informal leaders	34% < 15.000; 49% < 50.000; 11% < 100.000; 6% > 100.000.	63% Milano area; 11% Bergamo area; 9% Brescia area; 17% Monza and Brianza area.
Phase 3 (June – July 2021)	31	F = 29%; M = 71%	84% formal leaders; 16% informal leaders	38% < 15.000; 42% < 50.000; 13% < 100.000; 7% > 100.000.	68% Milano area; 10% Bergamo area; 6% Brescia area; 16% Monza and Brianza area.

*Note*: Interviews have been conducted at three points in time (Phase 1, 2 and 3) on the same sample of leaders (n = 42 at time 1; n = 35 at time 2; n = 31 at time 3; missing interviews are due to impossibility to reach the respondent or to role switch).

### Qualitative Data Collection

We conducted semi‐structured interviews with 42 participants, who played an active leadership role during the COVID‐19 crisis in the four selected areas. Specifically, we considered formal and informal leaders in charge of decision‐making, management, provision and/or coordination of services that were relevant for the functioning of the local communities during the crisis (mainly organizational and support services – i.e., social, welfare, food provision, support, safety, spiritual, transportation and logistics related services; e.g., Bonjean and Olson, 1964; Sancino et al., 2018). We did not interview any medical or health care professional involved in the sanitary and health emergency. We interviewed different categories of leaders, both with formal (e.g., directorship of a unit or organization) or informal (e.g., coordination responsibilities even in absence of formal hierarchical power) roles in the public, private and non‐profit sector, which allowed us to develop a broad overview of networking behaviours during the crisis. Following recommendations from previous research (Kilduff et al., 2008), the inclusion of both formal and informal leaders in the study design (described in detail in Appendix 1) helps disentangle the impact of formal structural arrangements and informal influence on the actors' networking.

#### Sampling

To select the interviewees, we followed Lincoln and Guba's (1985) recommendations for ‘purposeful sampling’. We started with an open call to a set of public, private and non‐profit institutions of different size and geographical location within the four provinces. The open call had a focus on ‘leadership in a time of emergency’ and was made available through detailed posts in dedicated WhatsApp and/or Facebook groups involving a representative number of local leaders and in other institutional portals. The excellent access to the research site was facilitated by the previous experience of the second author of this paper as a city leader in the same region. Through this open call, we recruited 18 participants. Then, we adopted a snowball technique, asking interviewees to suggest other leaders that they thought we should interview, rendering our theoretical sampling technique both deliberate and emergent (e.g., Dacin et al., 2010, p. 1399). We combined this procedure with processes of ‘theoretical sampling’, focusing on gathering data relevant to the theoretical concepts emerging from the ongoing investigation and from comparison across respondents (Corley and Gioia, 2004, p. 180). This method allowed reaching an evolving sample of respondents, with increasing focus on data that, despite the limited time horizon, enabled progressing towards acceptable levels of theoretical saturation (Glaser and Strauss, 1967).

We had access to an initial sample of 54 people. We decided to focus only on leaders working exclusively in the four selected areas (and not in neighbouring provinces) and with direct (formal or informal) influence on task management and service provision (and not, for example, with main or pure institutional role), which led us to retain a sample of 47 leaders. To allow comparability across interviews, in line with our arguments on leadership in a time of crisis, we further restricted the analysis to leaders who were directly involved in the management of services during the pandemic; moreover, we focused on organizational contexts with a clear relationship between leaders and followers. These specifications led us to retain interviews with 42 leaders. All interviews were conducted by phone or online platforms (Skype, Teams, or Zoom). For a number of respondents, due to confidentiality issues, we could only take extensive notes, including verbatim quotes from the interviewees, and then validated those notes with the respondents. Table I summarizes the final list of interviewees' categories and roles.

#### Semi‐structured interviews

Data collection consisted of three phases: we interviewed the same respondents during the first and the second wave of the crisis and in a follow up phase in a moment of relative non‐emergency. The interviews with the leaders lasted 35–75 minutes in the first phase (n = 42; on average, 48 minutes). The starting protocol was mostly standardized across respondents, with limited adaptation for hierarchical level and seniority of leadership, type of organization and geographical area. All initial interviews involved questions concerning (i) an introductory overview of the leader's job and professional role; (ii) the effects of COVID‐19 on the leader's job and role; (iii) the effects of COVID‐19 on the institutional, professional and interpersonal collaborations of the leader with other actors in the management of community services; and (iv) the effects of COVID‐19 on the leader's personal approach to role, interactions and networking, with focus on the evolution of the crisis and future prospects. During the first phase of the research, subsequent interviews with respondents became progressively more structured as the crisis evolved and themes emerged in the data, with the addition of questions on the effects of the lockdown on organizational functioning and collaborations, on the ways leaders subjectively perceived roles and relationships during the crisis, and on specific topics eventually mentioned by the respondent (Corley and Gioia, 2004). At the conclusion of the interviews, we provided respondents with an opportunity to give us feedback and recall any final thoughts.

The interviews in the second phase with the same respondents (n = 35; seven respondents were not reachable due to personal reasons or role change) served as follow‐up interviews aimed at eliciting the differences in the leaders' approaches to the crisis in the second versus the first wave of the crisis; and, more specifically, at grounding leaders' perspectives on networks, networking and personal reactions that emerged from the interviews conducted in the first phase. Interviews were structured around (i) the effects of the second wave on person, job and role; (ii) effects of the second wave on the personal reaction to the crisis; (iii) effects of the second wave on interpersonal and social interactions and networking; (iv) and effects of the second wave on the leader's personal approach to herself and to others. Interviews in the second phase lasted 25–40 minutes (on average, 31 minutes).

The third phase (n = 31; 4 respondents included in the first two phases were not reachable or switched role) served mainly as a follow up of the first two phases, with the aim to check patterns of stability and variability in personal reaction to the pandemic, network interactions and networking for the leaders involved in the study. The interview protocol was slightly different from the ones used in the first two phases and adapted to the specific moment in which data were collected. Questions focused mainly on (i) what the respondents learned from the crisis and what was the overall impact of the crisis on the person, job and role; (ii) on a summary of the experience of leaders, with focus on their interactions with others (followers, stakeholders and community) and on the relational approaches to their roles; (iii) on perceived changes in these dimensions compared to the period before the emergency, with emphasis on the distinct phases of the emergency and on the vaccination campaign phase. Interviews in this third phase lasted 20–45 minutes (on average, 33 minutes). We include the detailed interview protocols for all phases in Appendices B, C and D.

### Qualitative Data Analysis

As we collected the data, we started to analyse these data inductively (Gioia et al., 2013), following recommendations for naturalistic inquiry methods (Lincoln and Guba, 1985) and constant comparison techniques (Glaser and Strauss, 1967). In our view, generating theory and ‘doing organizational research’ are complementary processes (Glaser, 1978; Greenwood and Levin, 2006). Adhering closely to established techniques for theory building in qualitative research (e.g., Corley and Gioia, 2004), the coding analysis comprised several steps. First, from the raw interview data, we started identifying initial concepts associated with networking and grouped them in tentative categories (open coding). Conceptual coding used *first‐order categories*, identifying statements made (and repeated over time) by the participants, when possible, or a simple descriptive sentence. After categories were generated, we checked the data again to see which fitted each category. If the data did not fit well into a category, that category was changed or dropped (e.g., Dacin et al., 2010). Second, we ran axial coding, searching for conceptual relationships between categories, with the aim to integrate such categories into higher level, *networking actions*. We define networking actions as repeated patterns of relational activity manifested by the leaders across different answers and social situations. In a final step, we collapsed these networking actions into more theoretically and abstract *domains of networking*, which represent theoretically‐informed agentic repertoires underlying leaders' networking behaviours. Of note, we based our analysis mainly on data collected in the first two phases of data collection; the third collection served to validate the assumptions made on the previously gathered data, and to observe patterns of variability in networking actions. When an action emerged in the first two phases but was not traceable in the third phase, it was discarded by the final analysis. This re‐examination of the overall data served to test the fit of the raw interview material with the emergent actions. Networking actions and representative quotes are reported in Table II.

**Table II:** Findings of the qualitative data analysis: networking actions and representative data

Networking domains and actions	Representative data
*Networking domain: Dynamics in ego‐network structure*	
1. Network generation	
A. Seeking out new contacts.	A. ‘Building your own network has always been a priority for everyone in managerial roles, but never as much as in the pandemic. I see that managers who provide answer to the population are those who expand the size of their connections, creating new contacts and going out a lot to others to ask for advice, for help, for information, for resources. Since the beginning of this terrible emergency, I have spent considerable time to make new contacts, because I know that they can be useful. Whoever I talk to – I share my contact details and I ask them to do the same with me. It might seems useless, but is not. The opposite, I would say. If you need urgent advice or help, you can call them and they are really available. In this emergency, everybody makes herself available to help, with good spirit of service. But this is why expanding the size of your contacts is crucial to be an effective leader’. (Respondent 6)
B. Broadening the spectrum of the leader's network.	B. ‘Nothing like the lack of knowledge of the events generates fear and anxiety, because no solution is possible if you do not know how to provide the solution. I was lucky enough to have a variety of contacts in different institutions, which I mainly built in my previous managerial appointments, and which were extremely helpful in this circumstance. Medical specialists were giving me precise details of how the infection could spread and harm people, which categories it could harm the most, etc. They were giving me new knowledge as soon as they had new knowledge. In parallel, interaction with scientists of the regional and national offices helped me frame the possible institutional response in a more precise and straightforward way … The point here is to make my *rete di supporto* [support network] wider, more extended, more variegate. How to do it? The easiest way, and more helpful, was putting together these disconnected pieces of a puzzle to create a big mosaic, where fragments that are far from each other are all reachable’. (Respondent 20).
2. Network termination	
C. Dropping existing ties.	C. ‘To make new contacts requires energy, and there is no doubt that making contacts is important professionally each day of our work life, but this is also more important when there is a crisis that requires answers …. But the more you make contacts, the more you fill the energy space that you can give to your social life. So it is also important to cut contacts. It is difficult to say when. Maybe when they are not useful. Or sometimes there is no even a reason, you lose contacts just because there is no mutual interest to talk to each other. But I saw this happening a lot during this period. I cut off a lot of these contacts, mainly because I did not see the utility in this difficult moment’. (Respondent 34)
D. Cutting off parts of the leader's network.	D. ‘The real truth is that we cannot manage contacts that are not of help, in particular during an emergency so intense like this one. Coordination with partners that are not responsive, or partnership with other companies that do not have the same priorities can even be detrimental to our company's entrepreneurial and social mission. This is true for my company but this is also true for myself, because connection with these partners means connection with a lot of people. I know that this seems weird when I say it, “I interrupted my connections with them”, but in the very end it is exactly what happened. I avoided contacts with entire sets of acquaintances that represent, at least in this moment, more an impairment than a source of help. I do not know yet whether this will be temporary or not, but I realized that some relevant parts of our interpersonal life are not as good as we think, but we realize so only when there is a situation of emergency that urges us to think of the utility of connections. In normal life, this is something we barely do and, even when we do it, usually there is no consequence and things continue as they always did, even if it is highly inefficient’. (Respondent 41)
*Networking domain: Dynamics in network utilization*	
3. Network conflict.	
E. Looking for solutions through conflict with others.	E. ‘This was a big impact of COVID‐19 on our way to interact with others, and with others I include our colleagues and external parties and collaborators. Before the emergency, open debate was not very appreciated. Maybe it is cultural, here we still have this polished search for consensus. But it was also personal. Everything goes through consensus, decisions need consensus, and we all know that often this consensus was only a formal social norm just to give the green light to decisions – in the end, who is so brave enough to say “no” when you are the only one who is against a decision? But now, and let me say, finally, this has changed. People feel free to say no, and decisions often are the final moment of very open discussions, in which being confrontational not only is not forbidden, but I would rather admit it is also appreciated. Being confrontational, at least a bit, is a way to show that “I care, I want to make the best decision”, so it is not a conflict against somebody, nothing personal, but just a constructive way to be very direct, even maybe rude, but for a good end’. (Respondent 9)
F. Expressing emotions that can lead to interpersonal conflicts in decision‐making processes	F. ‘Looking back, we were all feeling scared, angry, fearful, and you could add a lot of these feelings to describe the situation. However, this was not as negative as it can seem at a first sight. For the first time maybe in our entire professional life, we were free to express the way we were feeling. “Free” to do it, without constraints, without formal roles, etc. I could tell others that I was angry about something without the risk of being perceived inappropriate, and it was the same for others. It is clear that this led to several outbursts, and also to open confrontation. I remember a situation in which maybe voices were a bit too loud. But, retrospectively, this was a cathartic moment that helped us to deal with the context. We were ourselves, we were doing our best in a terrible and unprecedented moment, and yes we were close to explode emotionally. But it was not just “I want to show I am the best”. Not at all. It was more, “I am close to explode because I want things to work and I am doing my best, so even if we are confrontational, even if we express anger and fear, it is because we want things to work”’. (Respondent 30)
4. Network deepening	
G. Enhanced investment (time and effort) in existing contacts	G. ‘I have no doubt that my connections with the rest of the team have become more frequent and intense, and that I talked in the team very often to people with whom I barely talked before. We feel the same, so talking is easier – we are not shy or blocked, not anymore. From my personal side, this is the result of a lot of time, attachment and attention to others in this troubled time’. (Respondent 14)
H. Investigating how to develop existing relationships in a more comprehensive way (for example, to find solutions to problems)	H. ‘Once you realize that you knew people for almost all your working life, but you never really had to work with them to solve important problems … Then, when it is the moment to harvest value from this relationship, you know that you have to do something different, in the way you manage the relationship with this person. With some people it is just matter to spend more time looking at the problems and looking for solutions. With other people, it is more an in‐depth reflection to answer this question: “How can I make my relationship with John [fictional name] useful in solving this issue?” It is always a matter of spending more time with the person, but also to build on her or his abilities to make a better use of it. It is something we never think about, in such an intense way, in the routine flow of activities, but in such a difficult context it was much needed’. (Respondent 25)
*Networking domain: Dynamics in network interpretation*	
5. Network teleology	
I. Focusing on the personal purpose of the relationship with others	I. ‘Most of our managerial and even institutional commitment involves interpersonal relationships. We spend a lot of our working days with colleagues and members of companies and other municipalities and institutions, but we have little or no attention to the scope, to the purpose of these relationships. There is always a big “Why” that we never investigate. Why do we have this connection? What is the purpose? I mean, not only the professional or institutional purpose, but also and maybe even more important, the personal purpose. How does it enrich my life, and my work? In terms for example of competences, personal growth, ability to solve problems? This moment of emergency was the chance to think about this “Why”. I shared this reflection with many colleagues, and they all agree that is very important to our work as coordinators in the communities’. (Respondent 10).
L. Understanding the (organizational and personal) scope of the tie by exploring the person beyond the role	L. ‘In normal times, we missed the chance to see the “human being” we were working with. We were close but distant, on a personal level. The work routine was prevailing and imposing the usual stuff, the same way of work … [Now, in a time of crisis] We are distant but close, and being together helped us understand that all this individual suffering is relevant to all of us – individually and collectively. There was a meaning in it, even if obscure and inscrutable. As single humans, we could grasp only a small part of it, but, together, we could feel it. And it was a source of strength that could contrast fear’. (Respondent 16)
6. Network re‐construal	
M. Reconsidering the opinion about potential ties (which can lead to new ties)	M. ‘Generally, we have the connections we need. We talk to the people who share with us tasks and responsibilities, we interact with people who are functionally assigned to us [as leaders], we report to the apical levels of our organizations. This common view is not equally true in this pandemic time. I, and many others, had the need to think about relationships that we did not have yet, but could be useful at that time, in that moment. How can I find the protection material that I need? How can I solve issues that I cannot solve with the connections I have now? So, you start thinking about connections that are not there, but are possible. People that are in the white book of the possible ties but that you never realized, or activated. Some are connections from the past, others are connections that you know you have but you never activated. So this re‐interpretation can help make the possible real and help face issues otherwise apparently impossible to solve’. (Respondent 40)
N. Re‐thinking the meaning of existing ties (which can lead to different nature of existing ties)	N. ‘It is clear that after you work with such a crazy intensity, you start seeing the relationships in a different way. You realize that the interpersonal resources can be different, in their intrinsic component, from the way you thought before. It is a matter of re‐thinking the ‘dimensions’ of these professional connections. What seemed to be just an institutional connection can become a reliable source of advice; what was just a relationship aimed at sharing information can become a useful connection to try gathering resources and coordinate activities. The real identity of these connections, as I said, not only depends on what we do together, but also on the way we see and experience the connections. If we change the way we interpret it, then the intrinsic component of these connection can change’. (Respondent 19)

### Trustworthiness of the Data

We followed Lincoln and Guba (1985) and Corbin and Strauss (2014) to take steps aimed at ensuring the robustness and generalizability of the inductive analysis. We aimed to reach conceptual density, developing concepts and relationships between the concepts in ‘great familiarity’ with the collected data, through an ongoing process of data collection and analysis that was interwoven with theory development (Glaser and Strauss, 1967). In the empirical context of our study, this was facilitated also by the structuring of data collection in different phases (see Table I and Appendix 1), which helped the reflective interplay between data collection, continuous data analysis and emergent theorizing. Notably, our conceptualization and operationalization of conceptual density differs from Geertz's (1973) ‘thick description’, in which the emphasis of data analysis was more on description of the ongoing findings than on abstraction and conceptualization. Constructs emerging from data collection and analysis enter analytically in our theorizing as conditions that enable further validation or change of the constructs themselves (Strauss and Corbin, 1994, p. 276). This allowed developing plausible relationships among concepts and sets of concepts, which enabled the discovery process of patterns of actions and interactions in the unfolding dynamics of the reported events. Grounding our analysis to the idiosyncratic social and historical conditions of our data analysis helped us to track ‘movement’ in our conceptual and analytical patterns (Dodier and Baszanger, 1997), which led us ultimately to analyse networking behaviours over time.

Specifically, during and after the course of data collection, validation consisted of inter‐rater reliability checks, reflecting memos and peer debriefing (e.g., Levitt et al., 2018). In each phase of data collection, to avoid subjective bias in the coding procedure, the two authors initially selected and independently coded a sample of eight to eleven relevant sections of transcripts from seven interviews (across the three phases), which were then discussed in detail to make sense of any possible ambiguity. Both authors then conducted independently a further round of coding with other 25 relevant sections of transcripts from ten different interviews. We calculated Cohen's Kappa in this subsample of material, which obtained a value of 0.84 (e.g., Fleiss et al., 2013). Any lack of agreement between the two coders was addressed through re‐analysis and discussion in the research team. Second, we used reflective memos throughout the data collection and coding processes, to keep track and reflect on the emerging understanding of the data. This helped the research team to confront on different insights retrospectively, thus making sense of any possible subjective bias in the coding process (e.g., Unsworth et al., 2018). In addition, we conducted peer debriefing, by engaging three outsider researchers, not involved in the research, to discuss and validate findings emerging from the data, thus providing insights and stimulating further questions.

### Inductive Findings on Leaders' Networking Actions

The central finding from the qualitative analysis is that, during the different phases of the crisis, leaders display combinations of the six networking actions summarized in Table II, which provides quotes from the interview material that lead inductively to the definition of the variables. These networking variables represent relational coping actions shown by leaders in reaction to the emergency.

The first variable emerging from the data is *network generation*, which encompasses actions oriented at either seeking out (new) contacts or, at an aggregate level, at broadening the spectrum of a leader's network. Network generation is at a maximum when a leader invests effort in generating a high number of new ties, reaching out to a high number of (new and existing) actors; or when the leaders extend the range of their network, by bridging their existing networks with other, previously non‐connected social groups. On the contrary, it is at a minimum when the leaders do not form new ties, thus restricting their network opportunities to the set of relationships already available; or do not extend the range of their local network to new groups, thus limiting the opportunities of expansion of knowledge search. The second variable is *network termination*, which refers to leaders' actions oriented at dropping existing ties (in particular, if such ties are perceived by the leader not useful during the crisis) or at cutting off parts of the network (again, losing contact with subgroups that are perceived not helpful in terms of reaction to the emergency). The first two networking actions, considered together, still follow a structural perspective that is almost primed in leaders' relational behaviours: they refer to leaders expanding or restricting their interactions patterns, either in numerical terms (number of contacts) or in the range of their networks (in terms of spanning between groups, or terminating bridging activity between groups).

Differently, the third and fourth variables pertain to actions associated with network utilization, i.e., the use that respondents make of ties they already have. The third action, *network conflict*, refers to the constructive abrasion and interpersonal frictions experienced by leaders in their interactions with followers. Through conflict, which implies open debate, confrontation and even constructive tension, leaders utilize their networks in search of solutions while facing emergent problems. Through conflict, leaders do not refrain, in a time of crisis, from showing their true selves, expressing (even negative) emotions that break formalized, structural barriers in their interactions with others. This variable is at a maximum when leaders fully express conflict and abrasion, engaging in open and constructive confrontation; whereas it is at its minimum when leaders refrain from it in their interpersonal relationships. The fourth variable, *network deepening*, encompasses the actions by which leaders invest time and effort in deepening their relationships with existing contacts, and in investigating how to make a better use of their contacts in search of solutions. Although both network conflict and deepening imply a certain degree of closeness between leaders and followers, they encompass networking actions that leaders undertake concerning the use of their networks, either in terms of experiencing conflict or deepening their (existing) ties in search of instrumental solutions. Their focus, therefore, is on network utilization, and not on the structural features of the network.

The fifth and sixth actions, different from variables referring to network structure and utilization, pertain to the domain of the subjective interpretation that leaders develop about their social relationships. Here the focus is not on the structural or instrumental approaches to networking, but on the personal meaning that leaders attribute and even re‐assign to their potential and actual relationships in a moment of crisis. Specifically, we label the fifth action *network teleology*, as it refers to the subjective emphasis of leaders in reflecting over and searching for the personal – or, in case of formal leaders, also institutional – purpose of their interactions with others; and to the leader's effort to understand the scope of their relationship with individual alters by exploring the other person beyond the institutional or managerial role. Teleology is at its maximum when the leader actively engages in this meaningful purpose‐searching effort; whether is at the minimum when the leader does not exert any energy in purpose‐oriented efforts, keeping the relational focus of her or his networking purely structural and/or instrumental. The sixth variable, which we called *re‐construal*, sees the crisis as an opportunity for a subjective re‐assessment, from the perspective of the focal leader, of the meaning of both potential ties (ties that could exist but where not yet forged or activated) and existing ties with others. From a construal perspective, the leader re‐constructs the personal interpretation of both the opportunity and utility of connections, resulting in the generation of new ties or in a change in the nature and use of existing relationships. Taken together, teleology and re‐construal represent variables that encompass networking actions associated with the domain of meaning, purpose and interpretation of social relationships.

### Connections between the Six Networking Actions and Previous Research

In Table III, we analyse the conceptual links between the six networking actions emerging from our qualitative data analysis and parallel constructs discussed by previous research, briefly outlining common trends and new insights. For what concerns the first two actions (network generation and termination), they are substantially in line with previous structurally‐oriented research. It is clear the link with prior work on churning/dynamics in ego's network structure (Vissa and Bhagavatula, 2012), which looked at tie creation (e.g., Tasselli et al., 2020) and at the dropping of existing ties (e.g., Dahlander and McFarland, 2013; Kleinbaum, 2018).

**Table III:** Inductive variables describing networking actions and connections with previous research

Networking actions	Connections between inductive findings and previous research	
	Consistency with previous research	New insights emerging from the data
*Networking domain: Dynamics in ego‐network structure*		
1. Network generation	It is in line with recent work on network formation, in terms of tie creation (e.g., Tasselli et al., 2020) and broadening of the range of one's local network (Soda et al., 2021), as a key change in a leader's structure over time (for a recent review, see Chen et al., 2022). Moreover, classic network research analysed structural change in the contingent aftermath of an organizational ‘jolt’, with specific focus on ego‐network change (e.g., Sasovova et al., 2010) and on change in structural configurations (Barley, 1986) and power distribution in organizations (Burkhardt and Brass, 1990).	Focus on the expansion of the leaders' personal networks to connect with external groups, though the involvement in the network of new individuals and new groups.
2. Network termination	It is in line with studies that have investigated, at the dyadic level, which ties people decide to retain and which ones to drop (e.g., Dahlander and McFarland, 2013; Kleinbaum, 2018). Studies have also analysed how the loss of brokerage positions can reduce bridging opportunities in one's network (Burt, 2002).	Leaders not only drop existing ties, but, in parallel, they also cut off entire subparts of their networks, for example when subgroups are thought not to be useful in providing information or support during the crisis.
*Networking domain: Dynamics in network utilization*		
3. Network conflict	Previous work has explored the extent to which managing conflicting relations among others can bring advantage to the leader who ‘plays conflicting demands and preferences against one another’ (Burt, 1992, p. 34). This intuition follows the classic insight that actors achieve autonomy by occupying positions that have conflicting group affiliations (e.g., Tasselli and Kilduff, 2018). Recently, this idea has been developed by research showing that actors can be creative by mastering diverse and even conflicting relationships (for a recent review, see Zhou et al., 2019).	The data show two insights previously not explored by research. First, our evidence at least partly rejects the assumption concerning the positional information advantage of disconnection (e.g., Burt, 1992). In our sample, leaders use conflict with existing (and often cohesive) contacts as a constructive way to elicit new ideas and come up with new solutions. Second, this activity often involves emotional work. Conflict is not a neutral relational action; it involves frictions and emotional expression during the crisis, which allows leaders and followers to engage in problem solving by using emotional expression to break structural barriers.
4. Network deepening	From a structural perspective, research looked at ego‐network density and closure, i.e., the depth or embeddedness of somebody's relationships in the network. It has been found that density of ties within an ego network affects knowledge transfer (Reagans and McEvily, 2003) and task mastery (Morrison, 2002). At the dyadic level, prior research looked at tie strength, showing that strong ties, network cohesion, and network range affect knowledge sharing (e.g., McFadyen et al., 2009; Tortoriello et al., 2012).	The concept of network deepening was not explored by previous research in a behavioural fashion. Our data show that managers alternate strategic and serendipitous investments in existing contacts to search for knowledge, solutions to problems and cues for decision making. This path does not consist necessarily of increased tie strength (broadly defined as enhanced frequency and emotional involvement in a relationship; e.g., Marsden and Campbell, 1984), but can also involve punctuated patterns in which leaders deepen the relationship when in need of help or advice, then weakening the connection when the perceived utility of the tie is lower.
*Networking domain: Dynamics in network interpretation*		
5. Network teleology	Although neglected by structural research, given its emphasis on relational regularities, the focus of meaning and purpose (e.g., Godart and White, 2010) has been peculiar to social network research since Simmel's (1950) attention to ‘network colouring’, i.e., the subjective lens by which people see their social interactions. This purposive view is also intrinsic to Jacob Moreno's (1941) intuition that social networks are ‘catalyzers’ leading otherwise ‘passive agents’ to goal‐oriented action.	Our data show that meaning and purpose represent a fundamental part of leaders' ties, in particular in a moment of crisis in which structural and organizational regularities are substituted by uncertainty. Leaders not only form new ties and deepen the connections with others, but they are also involved in subjective patterns of re‐interpretation of the purpose of the tie, aiming at making sense of the emergency. In this sense, our study provides inductive support to the concept of ‘locus of action’, i.e., the extent to which people exert agency by giving (purposeful) meaning to otherwise serendipitous connections.
6. Network re‐construal	In recent research, there is interest in a construal approach to social networks, defined as the extent to which ‘individuals' entire self‐regulatory system’ explains their social interaction choices and their returns from social interactions (e.g., Brands and Mehra, 2019). A constructivist approach has also been applied to structural research analysing how information is interpreted by those who receive it (Perry‐Smith, 2014).	Unlike previous research, which focused mainly on contexts of organizational stability, our research shows that the crisis is an opportunity to re‐think and re‐interpret the meaning assigned to potential or existing social relationships. Of note, and different from previous work – which treated structure and network interpretation as different epistemological domains (for a recent review, see Tasselli and Kilduff, 2021) – we find that the subjective pattern by which leaders interpret relationships can also have structural effects. Leaders can indeed decide to form, or activate, ties that were only potential, and they can also decide to change the nature of existing ties. Interpretive action might thus result in change in structural configurations.

For what concerns the two actions in the domain of network utilization, which we labelled network conflict and deepening, they entail more innovative conceptual insights. They complement and extend (for what concerns conflict) previous research that looked at the stresses and strains associated with group affiliation (e.g., Tasselli and Kilduff, 2018) and (for what concerns deepening) studies emphasizing the effects on the outcomes of tie strength (e.g., Morrison, 2002) and local network density (e.g., McFadyen et al., 2009). Of note, previous research mainly looked at the afore mentioned constructs from a structural perspective, thus neglecting the behavioural elements behind a leader's activation and use of the network. These behaviours can involve emotional work, in particular in a context of emergency such as the one described in the study. In this sense, emotional expression can contribute further to break structural barriers.

The conceptual contribution of this study is even more compelling for the last two dimensions, which we labelled network teleology and re‐construal, which pertain to the domain of networking as a meaning system through which leaders interpret and construe their social reality. The focus on meaning and purpose has been intrinsic to social network research since its beginning: for example, Simmel (1950) refers to the ‘colouring’ that people give to their ties, and Jacob Moreno (1941) refers to networks as ‘catalyzers’ through which people give meaning to relational action. However, this introspective lens has been traditionally neglected by structurally‐informed sociological research, and has surfaced only recently with the renewed interest of organizational network researchers on networks as ‘systems of meaning’ (e.g., Godart and White, 2010) and on construal as an interpretive lens to network agency (e.g., Brands and Mehra, 2019). Through our qualitative data, we contribute to this debate by showing that leaders are deeply involved in subjective patterns of re‐interpretation of their networks, and that the subjective pattern by which leaders interpret relationships also has structural effects. Remarkably, we found that leaders' interpretive action might result in change in the configurations of their networks.

### Cluster Analysis

After the qualitative data analysis that led to the emergence of networking actions, we performed cluster analysis to define configurations that allow categorizing leaders in groups, or clusters, based on common trends observed in their relational actions. The epistemological assumptions of our study – (i) the reliance on an idiosyncratic crisis that provides the opportunity to observe leaders' actions as relatively unconstrained from routine structures, and the (ii) consequent fact that the variables (actions) are grounded in our data – provide a solid foundation for conducting cluster analysis (e.g., Bensaou et al., 2014). By using data collected at three points in time over approximately an 18‐month interval, the goal was to observe temporal patterns of consistency and variability in repertoires of relational actions that aggregate into situationally‐contingent patterns of networking behaviours.

We followed a multi‐phase procedure to perform cluster analysis (e.g., Hennig et al., 2015; Romesburg, 2004). First, we re‐analysed the qualitative data to generate measures of intensity of the six networking actions. We categorized each leader, for each networking action, on a five‐point Likert scale ranging from 1 to 5, where the value of ‘1’ means that a specific networking action is at a minimum in that leader's behaviour, a value of ‘3’ means that the leader's approach to that action is neutral, and a value of ‘5’ means that the specific action features prominently in a leader's behavioural repertoire (e.g., Bensaou et al., 2014). In Appendix 6, we provide examples of quotes for the endpoints of each scale. In case a leader did not mention in the three interviews elements relative to a specific action, we did not attribute any score to the leader for that action, resulting in a missing value in the cluster analysis. This coding and rating procedure was conducted initially by the authors; to validate it further, we recruited two external raters with experience in qualitative research and, after we provided them a sample of quotes, we asked them to replicate the ratings. The interrater reliability was 78 per cent. In case of disagreement between the rating done by the authors and the rating provided by the external raters, we went back to the original quotes, re‐analysed the data and decided the final score (e.g., Kaufman and Rousseeuw, 2009).

In additional analyses, for each leader, we split the qualitative material in three samples associated with each phase of data collection and we checked, based on the intensity scale for each networking variable, whether leaders changed considerably their networking orientations over time. In four cases, we detected clear changes in network generation (for two leaders) and network termination (for other two leaders), with these leaders reducing the extent of their structural activity during the third phase relative to the previous two; because we concluded that this change was due to an exogenous change in the gravity of the pandemic situation, we still retained the values previously assigned. In any case, when replicating the cluster analysis without the data on these four leaders, results for the other leaders remained the same. For other seven leaders, we did not detect change in networking actions, but a general reduction in the intensity level of each action at time 3 relative to time 1 and time 2. Again, after reconsideration of the qualitative material, we concluded this was due mainly to the change in the pandemic situation across the phases. We discuss this evidence in the presentation of the qualitative findings.

Second, we included a list of associated variables that might be used to control for alternative explanations – beyond the behavioural one – for the categorization of leaders in clusters. Because we collected leaders' cross‐sectional ego‐network data at three points in time during each phase of the data collection, we included as associated variable the number of contacts in each leader's network (variable ‘*ego‐network size*’, as reported by the leader).^[^ ^2^ ^]^ In the same ego‐network survey, we also collected data on the strength of each tie, as perceived by the leader. The average strength value for each leader's reported ties (ranging from 1 to 5; e.g., Tortoriello and Krackhardt, 2010) was used to compute, for each leader, scales of ‘*ego‐network tie strength*’.^[^ ^3^ ^]^ (For more details on the measurement of ego‐network scales, see Appendix 7). We also included information on the leaders' organizational roles, including a dummy variable tracking whether the leader had formal (‘1’) or informal (‘0’) role (variable *leader*'*s formal role*), and – only for leaders with formal responsibilities – the number of employees/supervisees in their directing reporting network (variable *size formal network*). We included dummies to account for the geographical location of the leaders, in terms of *geographical area*, and for the *size of their municipality* (< 15.000 residents, < 50.000 residents, < 100.000 residents, or > 100.000 residents). Finally, we considered leaders' demography in terms of *gender* (‘1’ = female; ‘0’ = male).

**Figure 1:**
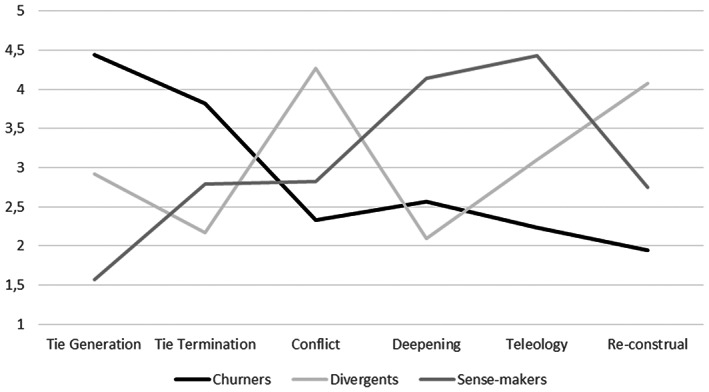
Cluster comparison on the mean levels of the six networking actions

Third, we conducted cluster analysis on the leaders' networking actions using STATA (e.g., Halpin, 2016). Hierarchical analysis, using Ward's algorithm and standardized variables, allowed us to generate agglomeration coefficients and dendograms, which helped us to conduct various analyses and develop considerations concerning the optimal cluster solution. We decided to retain three main clusters, which we labelled *Churners* (n = 16), *Divergents* (n = 12) and *Sense‐makers* (n = 14). Then, we conducted non‐hierarchical cluster analysis, generating k‐means coefficients for each cluster. We also performed t‐tests to check for statistical differences in networking actions between pairs of clusters; and ANOVA analysis for the networking actions and associated variables across the three clusters. The results of this analysis are reported in Table V. In Figure 1, we illustrate the mean scores for each cluster of leaders across the six networking actions.

Following standard practice for cluster analysis, we conducted additional tests, including the use of different clustering algorithms (e.g., Celebi, 2014), which provided confidence in the selection of the clusters. Furthermore, we conducted further qualitative member check inside and outside the contexts in which the respondents worked during the crisis. We interviewed five leaders (three in the same region and two outside the region) who were not in direct contact with the main respondents of our study. We showed the informants our basic findings concerning the analysis, asking for their feedback. The informants recognized these results as ‘plausible’ and did not signal any misleading argument in our conclusions.

### Findings: Leaders' Networking Behaviours during the Crisis

As soon as we started the interviews at the beginning of the crisis, we saw that leaders talked extensively of their relationships with other actors as a central part of their leadership role. For the vast majority of the interviewees, being a leader was perceived as a relational task, in which patterns of interaction with others were as important as their own actions. In the interviews, leaders often used heuristics that led them to depict their networks as a whole, using expressions like ‘*my network*’, ‘*my group*’, ‘*the web of my connections*’, or simply ‘*the gang of my angels*’, and even, with a musical metaphor, ‘*my Rat Pack*’. The structure of relational patterns tended on average to be described by the leaders, recalling Moreno (1941), as a *social atom*, in which the nuances of the interactions between alter and alter are often not intelligible. On the one hand, these ego‐centred heuristics limit our qualitative appreciation of what was going on in the network behind the leader's direct control; on the other hand, it allows us to unveil the behavioural manifestation of the network as reported by each individual leader. This is why we decided to focus on leaders' actions, with attention to networking (i.e., what leaders *do* when they interact with others) rather than on networks (i.e., the resulting structural configurations that emerge from ego's and alters' combined networking). What do leaders do when they interact with others? How do they behave in a situation of emergency? These are the main questions that we address with the identification of the three behavioural clusters that we describe here (see Table IV for relevant quotes on leaders' actions across clusters; see Table V for the ANOVA analysis and Figure 1 for a visual illustration comparing clusters in mean levels of networking actions).

**Table IV:** Sample quotes on the behaviours of churners, divergents and sense‐makers across networking actions

Cluster	Networking domain: Dynamics in ego‐network structure	Networking domain: Dynamics in network utilization	Networking domain: Dynamics in network interpretation
Churners	***High in network generation and network termination*** Quote: ‘When the emergency is at a peak, what shall I do as a leader? I shall be with others – of course online – more than before, as much as I can. I can have a look at my notebook and find contacts of people who can be of help: institutional actors, subjects from firms and non‐profit, other contacts that can provide answers and resources. The overriding rule? What kind of help can I find through these contacts, and of course, what kind of help can I give them? In this emergency, there is no time to know the person in a personal manner, there is no time to get “to know them well”. But, really, the emergency accelerated a lot our turnover in making and dropping contacts! Sometimes you make connections, sometimes it is time to forget this connection and move to the next one. And all of this is more frenetic than it was before. Is it a good way to exert leadership? I think so, because it can really help make my network bigger or smaller, depending on the leadership needs and on the organizational situations’. (Respondent 1)	***Neutral in network conflict and network deepening*** Quote: ‘For us, it was mostly about making connections that proved to be useful during the peak of the emergency. There was no time to reflect over the meaning and the learning arising from these connections. It was mostly about “doing”, and doing, in that context and in that moment, was being connected to people who could be of help’. (Respondent 41)	***Low in network teleology and network re‐construal*** Quote 1: ‘… That is why I really think that other colleagues, even in leadership positions, are a bit “slow” in their relationships … [Question about what the respondent means by “slow”] By “slow” I mean that they think too much, for example about how to deal with others, or why to interact with a certain person for a specific need. I do not understand why they do so. For me, it is definitely easier doing things rather than thinking too much … When I say “doing things” I mean talking to others, creating opportunities. For example, I called last week the central office and we got the contacts that helped us reach the producers in another region. Do you think that it would be possible if we spent too much time thinking of it, rather than acting?’. (Respondent 29) Quote 2: ‘Some relationships work. Some others do not. This happens at work very frequently. Ok, you can try to make it work. But there is no need to think too much of it, or to look at your relationship with others from another perspective, because 99 per cent of time still it does not work’. (Respondent 39)
Divergents	***Neutral in network formation, low in network termination*** Quote: ‘I would not say that I like arguing with others. Rather, the opposite. Arguing implies often emotional tension that drains energy. But sometimes it is the only way to generate new ideas, to look for solutions. You can talk freely, you can express yourself, you can understand the priorities for the other person, and try to come up with good solutions. Does it mean that I want to drop the connection with that person? No, not at all. An open confrontation sometimes, I would say most of the times, makes the connection even stronger, because we both know that we argue because we both care about the problems we want to solve, and once we find the solution, we know that our little argument was positive for both of us’. (Respondent 2)	***High in network conflict, low in network deepening*** Quote 1: ‘How do I generate ideas? Mainly “debating” with others. If done properly, this is the best way to deal with work connections in a time of crisis. You get the best that you can by being open, either when you agree with others or, even more, when you disagree. “Creative disruption” right? This is often a way to generate very good solutions in short time!’. (Respondent 40) Quote 2: ‘No, no, it is not about to get to know other people more, or better. Sincerely, I have to deal with enormous pressure. Who cares about focusing more on my relationship with Luc [fictional name]? The goal is to have things done … No, I am not worried that Luc [fictional name] gets angry at me. Indeed, I think he does not. He knows that even if we argue, it is because we are under pressure and is nothing personal against me or him or anybody else’. (Respondent 30)	***Neutral in network teleology, high in network re‐construal*** Quote 1: ‘A consequence of not being afraid of conflict? That then you start seeing relationships with others in different ways. “There is truth in war and in love”, an old proverb from my hometown said. I think it is a bit the same here, with my colleagues I mean, in particular during this pandemic. When you are open and ready to have arguments, then you start seeing the relationship from a different perspective. Even if you do not want it, the conflict creates a different image of that person in your eyes, and you cannot ignore it. So, also the relationship can change’. (Respondent 37) Quote 2: ‘I could not get what I needed [for the community]. It was evident. So what did I do? I looked at things differently. It is like when you are lost in a forest, it is dark, and you realize that the “Map” app in your phone is there not only to help you driving. You can use it in a different way, for example to get out of the forest. It is almost the same with people. You see in these difficult moments that people you did not see for a while, or who were not even in your network, can become very important in this different circumstance. It is mainly about looking around myself and looking at other people from a different perspective’. (Respondent 12)
Sense‐makers	***Low in network generation, neutral in network termination*** Quote: ‘My approach is very clear: first let us try to understand our people, then let us go to others. I am sometimes criticized for this clique‐like approach. But it is nothing against the external world, or against making new acquaintances. I am not an exception. But I see a lot of superficiality; I do not agree with colleagues whose priority seems making their web of connections bigger and bigger. And then, are you sure that you work better, especially during an emergency, when you deal with hundreds of contacts? How much time can you spend with each of them? How well can you get to know them? I mean, personally? I know my approach might seem quite strange in this hyper‐social collective mood, but I believe in getting to know people when I deal with them’. (Respondent 33)	***Neutral in network conflict, high in network deepening*** Quote: ‘I am not a hugely social person, you know what I mean. I still believe we need to have the time to focus on the colleague we talk to, to understand her or his ideas, to generate ideas by knowing each other not superficially, but spending the due time, attention, intelligence to understand what the other person thinks. I often find this investment in the relationship the best way to create positive working interactions with colleagues … No, this is not possible with everyone, I know, but at least I try. I would say, it is my way to approach others, irrespective of whether it will work or not. It is my approach to relationships, at work and, even if it is off topic in this discussion, also in my life’. (Respondent 31)	***High in network teleology, neutral in network re‐construal*** Quote: ‘I remember the day all started. I got a call at 7 am, the local hospital was getting full. It was like when you open a Pandora's Box and things cannot go back as they were before. I lived that experience with my co‐workers, with the other institutional actors, with my community. But so a question got stuck in my mind. What does it mean? Not just the events, but the true sense of my interaction with others. What is the inner purpose of these interactions? Why were we there together? With Gianni [fictional name, a mayor] we had a strong relationship because we had to help our community. With Luca [fictional name, an entrepreneur] we had to organize an operative response. This was the terrible but unique chance to get to know why I had a tie with all these people, something I never had the chance to think about, but that was so compelling during the crisis.’ (Respondent 10)

**Table V:** Means, standard deviations, and statistical differences among clusters in networking actions and associated variables

	Churners		Divergents		Sense‐makers		F
	Mean	SD	Mean	SD	Mean	SD	
**Networking actions**							
Generation	4.44	0.63	2.92	1.00	1.57	1.41	59.24**
Termination	3.82	0.54	2.17	0.94	2.79	0.58	20.64**
Conflict	2.33	0.72	4.27	0.90	2.82	0.60	21.96**
Deepening	2.57	0.85	2.09	1.22	4.14	0.36	20.69**
Teleology	2.23	0.73	3.1	0.88	4.43	0.51	33.82**
Re‐construal	1.94	1.06	4.08	1.38	2.75	0.45	14.61**
**Associated variables**							
Ego‐size T0	14.73	7.11	13.55	3.75	13.42	5.27	0.22
Ego‐size T1	18.4	8.45	11.67	2.61	10.64	3.67	7.79**
Ego‐size T2	13.5	4.52	11.11	2.62	10.18	2.82	2.85†
Ego‐size T3	12.75	2.92	11.57	3.82	11.8	4.1	0.31
Ego‐strength T0	3.17	0.68	3.18	0.64	3.59	0.63	1.36
Ego‐strength T1	3.53	0.64	3.5	0.48	4.08	0.49	4.55*
Ego‐strength T2	3.61	0.71	3.5	0.66	4.14	0.51	3.05†
Ego‐strength T3	3.58	0.79	3.69	0.80	3.56	0.53	0.08
Leader's formal role	0.81	0.40	1	0	0.64	0.50	2.84†
Size formal network	9.88	6.47	13.83	4.84	8.14	7.24	2.70†
Geographical area	1.19	1.42	0.58	1.08	0.57	0.85	1.36
Municipality size	0.88	0.96	1	0.95	1	1.18	0.93
Gender	0.31	0.48	0.42	0.52	0.29	0.47	0.26

*Note*: N = 42 for networking actions and associated variables. Due to missing data, for Ego‐size n = 38 (T0), 41 (T1), 34 (T2) and 29 (T3). For Ego‐strength, n = 37 (T0), 40 (T1), 34 (T2) and 29 (T3).

^†^ < 0.10;

* < 0.05;

** < 0.01.

### Churners

#### Networking behaviour

Churners have already been described by previous conceptual and empirical research with focus on interpersonal and intra‐organizational network dynamics (e.g., Sasovova et al., 2010; for a recent review, see Chen et al., 2022). Conscious of the limited novelty of the contribution provided by the analysis of this cluster, we still illustrate the actual behaviour of these leaders, who are particularly active in forming and dissolving ties with followers and other stakeholders. For Churners, the crisis is a powerful jolt that activates networking. Looking at the six actions that emerge from our inductive analysis (Table II), these leaders display high levels of network generation and network termination (see mean values in Table V). The emphasis of their networking is mainly structural: they orient their relational behaviour towards either developing new connections or dropping connections. For example, Marc (fictional name), board member of a municipality with responsibility for local task coordination during the crisis, described this process of tie formation and termination as an inherent part of his leadership duties (also in Table IV).

‘When the emergency is at a peak, what shall I do as a leader? I shall be with others – of course online – more than before, as much as I can. I can have a look at my notebook and find contacts of people who can be of help: institutional actors, subjects from firms and non‐profit, other contacts that can provide answers and resources. The overriding rule? What kind of help can I find through these contacts, and of course, what kind of help can I give them? In this emergency, there is no time to know the person in a personal manner, there is no time to get “to know them well”. But, really, the emergency accelerated a lot our turnover in making and dropping contacts! Sometimes you make connections, sometimes it is time to forget this connection and move to the next one. And of all this is more frenetic than it was before. Is it a good way to exert leadership? I think so, because it can really help make my network bigger or smaller, depending on the leadership needs and on the organizational situations’. (Respondent 1)

Churners show high levels of network generation and termination, but also low levels of the two interpretive networking variables emerging from our analysis, i.e., teleology and re‐construal (see ANOVA analysis in Table V). Churners are not particularly involved in thinking about the meaning, interpretation and purpose of their connections. For them, action is reified in structure – their behavioural pattern is manifested in the active turnover of their connections. This emerges from several interviews, including the one with a local entrepreneur in the field of mobility and transportation:

‘I can easily connect with many others, I can easily talk to them, create a contact, build a relationship that can help us solve daily issues at work, especially in this troubled moment. But honestly, that does not mean that I need to think too much about it. It is the way I am, for me it is very natural. It is a practical way to deal with problems. I am a “doer” and, I assume, this is what a leader should be good at’. (Respondent 41).

For Churners, the prevalence of tie creation and tie termination is not (per se) a strategic behaviour; rather, it is a manifestation of their relational self, a ‘*very natural*’ expression, as the respondent said, of their leadership style. This underlying behavioural element, for many Churners, was activated by the pandemic, which served as a jolt empowering their (often hidden) relational proclivities, making them more agentic in using structural behaviour to ‘*empower their leadership position*’. This point emerges clearly in the interview with a manager who had responsibility for the emergency unit of a group of municipalities in the region:

‘When the situation was normal, I mean before the viral emergency, I often had no time nor possibility to interact with others showing who I am … the degree of routinization of work is so high that sometimes you feel like a robot. You do “things” but in the end “things”, I mean the tasks, the workflow, those “things” make you do what they want. You know right? You do what the organization has already planned … But now it is different. I can take the lead and make the connections that I think are relevant. I am free in dealing with others – in contacting new people or not contacting usual subjects – and I feel, to some degree, empowered in my leadership position’. (Respondent 36)

#### Network structure

Not surprisingly, the self‐reported ego‐network size of Churners during the first phase (T1, winter and spring 2020) of the emergency (M = 18.4; SD = 8.45) was higher than the size of the ego‐networks of Divergents (M = 11.67; SD = 2.61) and Sense‐makers (M = 10.64; SD = 3.67). As shown by the ANOVA analysis in Table V, this mean difference in ego‐network size at T1 among clusters was significant (F = 7.79; p < 0.01). On the contrary, there was no mean difference in ego‐network size among clusters at T0, i.e., before the starting moment of the emergency, as recalled by the leaders. Consistent with our interviews, this suggests that, in a routinely phase of organizational life, the network size of Churners was not necessarily bigger than the network size of other leaders. Interestingly, during the second wave of data gathering (T2, late 2021), the still significant difference in size between clusters was reduced compared to T1.

#### Temporality

Did the pandemic shock activate the propensity of churners to engage in network turnover? Or, on the contrary, the churning behaviour triggered by the pandemic is bound to vanish when the pandemic is over? Qualitative evidence collected at time 3 (June – July 2021) suggests a more nuanced possibility: the pandemic awakens the likelihood of churning leaders to generate and terminate ties. However, the bureaucratic pressure exerted by organizational routines on leaders' networking behaviour ‘*strikes back*’ when the peak of the emergency is concluded. This intuition popped up from several interviews across clusters, and is expressed here by a municipality manager with responsibility for the overall coordination of the services to the community.

‘I always have in mind the title of that Star Wars movie: “The Empire Strikes Back”. Of course this is a metaphor. But you know what? This is how bureaucracy works. It seemed to be less oppressive during the moment of the emergency, because in that moment I could really feel the possibility to make a difference as a leader, making new contacts, creating opportunities. This is who I am! My way of leading others! But, then, formal procedures, task dependence, and any kind of formalization goes back to its natural oppressive power. And, suddenly, I feel pressure to go back to my routine, as if nothing happened. Can we resist to that? Is there a way?’. (Respondent 29)

### Divergents

#### Networking behaviour

For some leaders, the pandemic is not about changing the composition of their networks; rather, it is a chance for altering the management of existing connections, either in terms of activating open confrontation and even conflict with their acquaintances as a way to react to the crisis, or in terms of re‐interpreting and re‐construing the nature of their interactions with others. These are the Divergents – leaders who show, among the six networking actions elicited by our inductive study – high levels of network conflict and high levels of network re‐construal (see Table IV for relevant quotes). Whereas the networking focus of Churners was mainly associated with the structural domain of networking (network generation and termination), the behaviours of diverging leaders involve actions associated with both the network utilization (conflict) and the network interpretation (re‐construal) domains. Action and interpretation are oriented, for those leaders, towards managing their existing networks, with little or no structural implications. Conflict is not manifested through a high turnover of relationships – on average, divergent leaders do not drop ties frequently, as shown by the low levels of network termination (see Table V). Their behaviour is rather manifested in open confrontation that leads them to reconsider their opinion about acquaintances or to look at colleagues in different ways. This frank and even confrontational approach is captured well by a municipality board member with responsibility for the overall provision of services to the person (quote also in Table IV).

‘How do I generate ideas? Mainly “debating” with others. If done properly, this is the best way to deal with work connections in a time of crisis. You get the best that you can by being open, either when you agree with others or, even more, when you disagree. “Creative disruption” right? This is often a way to generate very good solutions in short time!’. (Respondent 40)

As suggested by this quote, and by interviews with other respondents, confrontation is often goal‐oriented. Diverging leaders engage in tension with others as a way to gain resources that can help them react to the emergency. Despite their openness towards open dialogue and debate, Divergents do not invest particular time, effort or energy in deepening their relationships with co‐workers – something that makes them different from Sense‐makers. This is reflected by their low average level of the network deepening variable (Table V). One reason for this finding can be found in the self‐serving nature of their ties: they do not fuel confrontation to get to know the other person better, but to find – through conflict and abrasion – solutions to urgent problems that require open debate and the removal of role‐associated barriers between leader and followers. The responsible of a non‐profit association providing care to homeless people remarks this point in a key moment of the interview.

‘No, no, it is not about to get to know other people more, or better. Sincerely, I have to deal with enormous pressure. Who cares about focusing more on my relationship with Luc [fictional name]? The goal is to have things done … No, I am not worried that Luc [fictional name] gets angry at me. Indeed, I think he does not. He knows that even if we argue, it is because we are under pressure and is nothing personal against me or him or anybody else’. (Respondent 30)

Despite the lack of investment in network deepening, conflict is still associated with high levels of network re‐construal. Tensions and conflicts, as suggested extensively by previous research (e.g., Ingram and Zou, 2008), are not neutral when we talk of work‐related relationships. Thus, for Divergents, the crisis is an opportunity for de‐freezing the routinely meaning assigned to their relationships, both in terms of reinterpreting the nature of existing connections, or seeing unacquainted people from different perspectives. We choose the words of an entrepreneur in the catering industry to illustrate this reflection.

‘A consequence of not being afraid of conflict? That then you start seeing relationships with others in different ways. “There is truth in war and in love”, an old proverb from my hometown said. I think it is a bit the same here, with my colleagues I mean, in particular during this pandemic. When you are open and ready to have arguments, then you start seeing the relationship from a different perspective. Even if you do not want it, the conflict creates a different image of that person in your eyes, and you cannot ignore it. So, also the relationship can change’. (Respondent 37)

#### Network structure

There was no significant difference in the ego‐network composition of Divergents (M = 13.55; SD = 3.75; see Table V), compared to the other two clusters of leaders, before the emergency (T0). During the crisis, Divergents displayed a remarkable stability in network size across the three phases of data gathering (with a mean value of ego‐network size = 11.67 at T1, 11.11 at T2 and 11.57 at T3). This is consistent with evidence emerging from qualitative analysis of little or no structural implications of their networking behaviours.

#### Temporality

Was the openness to confrontation of Divergents activated by the pandemic? And, did it fade away as soon as the pandemic seemed to be less severe (i.e., at T3)? As discussed in relation to the Churners, the answer to these questions seems again to be positive. There is consistency across interviews conducted during the third phase that the attitude towards conflict was predominantly associated with the emergency phase of the pandemic – a phase in which the emergency itself represented the opportunity for engaging in abrasive behaviour. Remarkably, when the emergency peak was over, leaders found themselves uncomfortable with engaging in conflicting behaviour, partly because of personal awareness of the situational change, partly because other actors urged them to change their approach to social interactions. This point is discussed by the city manager of a small town.

‘They looked at me like an alien. At the beginning, it was hard to conform. Come on, this was the same kind of answer I was giving to the same people just one month before that meeting. But, I realized it, the atmosphere was different. Less tension, less urgency, less likelihood to be so open towards others. The old habits associated with our formal roles, our hierarchies, all our routinized practices, were back. Nobody said that they had to be back, it just happened. But I felt so uncomfortable with my behavior, that I did not say anything for hours. Silence was the best way to avoid being perceived by others as non‐appropriate’. (Respondent 4)

### Sense‐Makers

#### Networking behaviour

Churners engage in structural behaviour – creating and dropping ties. Divergents are keen to abrasion and open to re‐interpret the nature of their relationships. For Sense‐makers, the third cluster emerging from our analysis, the pandemic is instead the opportunity for investing more time, energy and interpretive effort in ‘getting to know better’ their acquaintances – something manifested in the high levels of network deepening and network teleology (see Table IV for relevant quotes). Simmel (1950) noted that people tend to provide an ‘individualistic colouring’ to their connections: there is a dimension of networking that pertains to seeing others as part of a common destiny, to searching for the inter‐subjective meaning of social interactions. This is what sense‐makers do. Their structural change, captured by the churning variables, is limited – their level of network formation is low, whereas their level of network termination is neutral (see Table V). When the crisis hits hard, they do not search for new ties that can solve the many organizational problems. They prefer to dig deeper in their existing relationships, investing in ties they already have. This point is expressed by a public manager coordinating the local unit for mobility and transportation (quote reported also in Table IV):

‘I am not a hugely social person, you know what I mean. I still believe we need to have the time to focus on the colleague we talk to, to understand her or his ideas, to generate ideas by knowing each other not superficially, but spending the due time, attention, intelligence to understand what the other person thinks. I often find this investment in the relationship the best way to create positive working interactions with colleagues … No, this is not possible with everyone, I know, but at least I try. I would say, it is my way to approach others, irrespective of whether it will work or not. It is my approach to relationships, at work and, even if it is off topic in this discussion, also in my life’. (Respondent 31)

Interestingly, the need for depth of Sense‐makers is not just instrumental, i.e., oriented at solving problems or at getting things done. For these leaders, the crisis is the chance to see the *alter* as an individual beyond the network, to search for the underlying purpose of work interactions. This is why Sense‐makers are high in teleology: they interrogate themselves on the ‘why’ of network connections – something almost absent in the organizational social network literature,^[^ ^4^ ^]^ but that emerges quite clearly from our interviews, and in particular from what a municipality CEO said.

‘Most of our managerial and even institutional commitment involves interpersonal relationships. We spend a lot of our working days with colleagues and members of companies and other municipalities and institutions, but we have little or no attention to the scope, the purpose of these relationships. There is always a big “Why” that we never investigate. Why do we have this connection? What is the purpose? I mean, not only the professional or institutional purpose, but also and maybe even more important, the personal purpose. How does it enrich my life, and my work? In terms for example of competences, personal growth, ability to solve problems? This moment of emergency was the chance to think about this “Why”. I shared this reflection with many colleagues, and they all agree that is very important to our work a leaders in the communities’. (Respondent 10).

#### Network structure

Not surprisingly, the behavioural tendency of Sense‐makers towards network deepening is reflected by their average measures of ego‐network strength (Table V). Although the average tie‐strength measures recalled by sense‐making leaders (M = 3.59; SD = 0.63) were not statistically different from those reported by Churners (M = 3.17; SD = 0.68) and Divergents (M = 3.18; SD = 0.64) at T0 (i.e., before the pandemic), their values increased substantially at the beginning of the emergency (T1), showing for sense‐makers (M = 4.08; SD = 0.49) levels that were higher and statistically different from those of the other two clusters (Churners, M = 3.53; SD = 0.64; and Divergents, M = 3.5; SD = 0.48). We observed the same tendency during the second peak of the pandemic crisis (T2), but, remarkably, not at T3, when the mean value of ego‐network strength fell for Sense‐makers (M = 3.56; SD = 0.53) to levels that were very similar to those reported at T0, without statistical difference with the values claimed by Churners and Divergents.

#### Temporality

Again, this numerical evidence, associated and strengthened by qualitative evidence emerging our study, seems to validate the reflections on temporality discussed in relation to the other two clusters. For Sense‐makers, the pandemic activated a propensity towards deepening and teleology, something that several respondents confirmed to be part of their inner networking identity. This is expressed in a remarkable way by one of the coordinators of a multi‐sport club directly involved in the provision of local welfare.

‘For me, it was like finally discovering myself. All this attention to others, to the meaning of what we feel with others [emphasis given by the respondent], was always there, I mean, in my heart. But normally there is no way to let it emerge. Now, in this paradoxical situation, this is what is good in all this nightmare. This is who I am with others, I know it’. (Respondent 35)

However, the expression of this behavioural dimension was deemed to be more difficult when the emergency was at least partly alleviated. Respondents did not hide their behaviours, but they started feeling pressure to conform to social norms in which relationships are codified in formalized schemes of action that hamper the expression of their inner relational identities. This is the point made by the coordinator of an association giving services to people affected by disabilities.

‘What a sense of discomfort! We got used to know each other better, in those terrible days. We were so close, without all these ridiculous formalities, and I really had the feeling that working together was also a way to face together the huge emotional burden we were experiencing. But then, what happened? Abracadabra. When the situation went back to a sort of normal, I had the impression that people felt the need to switch back to the way we were interacting before the emergency. Again routines, rules, distance. I do not know why it happened, nor what or how they felt about it. But I feel uncomfortable, because for me things are not the same way they were the day before this big thing happened’. (Respondent 32)

## ADDITIONAL ANALYSIS

### Formal and Informal Leadership

In our sample, we included both leaders with a formal role (n = 34), and emergent leaders with a clear and recognized informal role in their organization or local community (n = 8). Did we find any difference in networking behaviours across clusters between formal and informal leaders? Analysis of the qualitative material showed overall consistency across formal and informal leaders. However, we found two interesting insights, which can be starting points for future investigation. First, formal leaders did not reveal any issue of legitimacy in their interactions with followers and stakeholders. Their prescribed, organizationally‐legitimized role gave those leaders the degree of authority and trust that was needed to engage in networking behaviour with followers and stakeholders during the crisis. We found this pattern for the three clusters, but especially for diverging leaders: for them, abrasion and even conflict were facilitated by the psychological protection given by their formal role. This insight emerged prominently in the interview with a municipality board member with responsibility for the overall provision of services to the population in the local community:

‘Having an official, formalized responsibility was fundamental, in particular in the first phase of the emergency, in dealing with others. Imagine that you have to reject another person's idea. This person would think. “Who are you to reject my idea?”. “What is the source of your authority?”. In our context, these questions can challenge your role. In this case, my authority within the municipality made much easier my role in engaging with others’. (Respondent 40)

Second, we found idiosyncratic patterns for informal leaders who displayed sense‐making behaviour. Sense‐making implies deep involvement in meaning generation and in teleology. Given the lack of formal organizational authority, the leadership of informal leaders tends to be ‘embedded in social ties’, such that their leadership style resides *in* the dyadic, informal ties that they develop and entertain with other actors (e.g., Carter et al., 2015). Remarkably, informal leaders tended to form connections with others mainly through personal contact (for example by giving advice or psychological support, or by helping others in problem solving) rather than via formally prescribed ties. The involvement of personal connections facilitated the sense‐making propensities of these informal leaders, who engaged in dyadic connections with followers and stakeholders in a more self‐reflective way. This evidence emerged from several interviews, including the one conducted with the informal coordinator of a non‐profit association aimed at offering free education to the population.

‘Leadership is not about hierarchy, or about telling people what they have to do because you are the “boss”. Leadership is about building deep connections with others, supporting others, being there for them, being of help and learning to recognize what they think and, even more, what they need. If you deal with others in a personal way, they do not care whether you have organizational responsibility or not. And then it is easier to establish a personal, caring, open and meaningful relationship with them’. (Respondent 42)

## LEADERS' BEHAVIOURS IN THE EYES OF THEIR FOLLOWERS

In this study, we focused on leaders' perceptions of their interactions with followers. The reverse question is interesting too: How do followers describe and assess leaders' networking behaviours? To give an explorative yet preliminary answer to this question, we conducted an additional, post‐hoc qualitative data collection involving short, semi‐structured interviews with 21 followers working with 19 of the leaders included in the study (interviews ranged from 11 to 23 minutes). We followed a semi‐structured protocol in which we mirrored, from the perspective of the follower, the same categories of questions asked to the leader: we asked followers to recall the leader's behaviour during the crisis, and the leader's interaction style with the respondent and with other actors (either followers, stakeholders or other leaders). The protocol is included in Appendix 5. After the interviews, we asked external raters (the same who previously assessed the self‐reported leaders' behaviours) to categorize the selected leaders, based on their followers' qualitative descriptions, on the six networking actions on the same, previously used five‐point Likert scale. Doing so, the raters gave each leader for each networking action two separate scores (each ranging 1 to 5): (i) the score based on the qualitative material collected through interviews with the leaders themselves (previously reported in Table V); and (ii) the score based on the interview(s) in which the follower(s) described the focal leader. The comparison of leaders' scores for each networking action based on the interviews (i) with the leaders themselves and (ii) with the followers reported a high degree of correlation for the networking actions of Churners (0.70; p < 0.01) and Divergents (0.68; p < 0.01). For Sense‐makers, this value was still significant but lower (0.35; p < 0.01). We analysed the data more inductively and found that the relational actions of Sense‐makers (in particular, actions aimed at deepening and finding meaning in social relationships) were relatively less visible to external observers (in this case, the followers) than the actions of the other two clusters of leaders. While ‘churning’ and ‘divergence’ are relatively visible networking behaviours of leaders,^[^ ^5^ ^]^ ‘sense‐making’ seems to (at least partly) escape the abilities of followers to understand and react to their leaders' behaviour. We found quite remarkable the insight provided by the employee working with an entrepreneur involved in the social care sector:

‘Sometimes it takes time, and some effort, to understand what he has in mind. He talks with you with great calm, even if the situation is compelling. He wants to know what you think of it [a specific situation], and this is sometimes puzzling, because I feel a sort of pressure to tell him: “Come on, let's do it. We have no time to think further of it”. But, most of the times, I do not say anything. Indeed, he has a clear ability to make good decision after these deep conversations, even if, I have to admit, I barely detect what he really thinks’. (Follower of respondent 33).

Sense‐makers navigate personal connections as a canvas to recognize the ‘*big why*’ (quote from respondent 37) of the surrounding events; they try to understand the ‘*big purpose of our being together*’ (quote from respondent 33). Their behaviour consists of introspection and often involves ties in which the leader uses the relationship with others as a ‘*mirror*’ to deepen her or his awareness and knowledge of reality. Behaviours have implications for the alter‐perceived agency of the leader. If the actions of Churners and Divergents – albeit very different from each other – are both quite visible to external actors (Churners form and dissolve ties; Divergents actively engage in debate and even conflict with others), this alters' acuity is more fleeting and ephemeral for those actors who deal with Sense‐makers. The agency of sense‐making leaders is perceived as ‘*more distant*’ (quote from follower of respondent 31), almost ineffable, unless is materialized in concrete actions that give followers a clear direction. We only have preliminary data to support this intuition, although at least three interviews clearly converge on this point. Here a quote from an interview with the follower of a manager of a non‐profit organization providing welfare and social services to the population.

‘Anna [fictional name] is a great leader … There is an aura of mystery around her leadership style. There are situations in which she talks with you and we are a mirror to each other. You know what I mean, right? We are not so focused on ourselves, but we try to understand each other's motives. Of course, this makes more difficult to understand what she thinks and how she makes decisions. This leadership style is demanding for her and for us; it takes time to get to know each other well, and to realize our thoughts and our goals. But, when she makes the decision, the decision is always clear and correct. And this reduces the pressure on the team’. (Follower of respondent 21).

## DISCUSSION

We conducted multi‐phase qualitative research and cluster analysis on a sample of 42 leaders in four of the most affected provinces in Northern Italy during the COVID‐19 pandemic. Through semi‐structured interviews, we investigated behavioural patterns of stability and change in leaders' networking. From our data analysis, six networking actions emerged, which represent the menu of behavioural repertories enacted by leaders during the crisis. Cluster analysis allowed us to categorize leaders in three groups – Churners, Divergents and Sense‐makers – distinguished by the behavioural networking approach to the emergency. Despite several limitations, our research allows a better understanding of the relationship between leadership and networking in the context of organizational crisis and disruption.

### Contributions to Theory and Future Directions

This study makes a distinctive contribution to theory and research on the micro‐foundations of leaders' networking behaviour in the context of organizational crisis (e.g., Tasselli et al., 2015). Our findings expand on the focus of previous research on leaders' network processes (e.g., Balkundi and Kilduff, 2006; Carter et al., 2015), bringing idiographic attention to networking as a situationally‐contingent behavioural process. Remarkably, the COVID‐19 crisis represented an epistemological jolt, i.e., an exceptional circumstance altering routinely networking patterns, giving the opportunity to investigate the nexus between leaders' action and interaction. In this idiosyncratic situation, leaders' reactions to ecological stimuli tended to coalesce in a process of emergence of ‘intra‐individual patterning of behaviors’ (Allport, 1937).

The evidence emerging from our study anticipates new questions for future research concerning the analysis of the antecedents of leaders' networking behaviours, and the implications of such behaviours for leaders' agency and for organizational coordination and functioning. What explains the emergence of networking behaviours? Do underlying leaders' characteristics affect the behaviours that leaders manifest in a situation of crisis? Answering these questions requires bridging nomothetic and idiographic approaches to behaviour. There is evidence, for example, that high self‐monitoring leaders (i.e., individuals who can flexibly adapt their self‐presentations across social situations) are more likely to gain co‐workers' trust in contexts characterized by the need to develop diplomatic skills and cognitive acuity in aligning to others' motives (e.g., Tasselli and Kilduff, 2018). There is also evidence that high self‐monitoring individuals engage in a high turnover of interpersonal relationships in organizational contexts characterized by a reshuffling of interpersonal ties, coming to occupy go‐between positions at the crossroads between separates social groups (e.g., Sasovova et al., 2010). In our taxonomy, these network characteristics of high self‐monitoring leaders can be found both in Sense‐makers, who spend time and effort in deepening their relationships with others, and in Churners, who are active in forming and dissolving ties. Despite the lack of data on leaders' personality traits in our sample, future research should analyse whether inter‐individual differences in traits are reflected in the intra‐individual behavioural patterns manifested by distinctive leaders. Both Churners and Sense‐makers, for example, could display high levels of self‐monitoring, but, from an idiographic approach, the same trait would be associated with idiosyncratic differences in networking behaviours between clusters.

This intuition calls for more research on the source of agency in explaining leaders' networking behaviour (e.g., DeRue et al., 2015; Tasselli and Kilduff, 2021). Are leaders strategic and goal oriented in their relational approach to the crisis (i.e., is the source of agency from without), or do they simply follow their inherent behavioural propensities (i.e., is the source of agency from within)? We did not find any conclusive answer to whether leaders strategically use networking to deal with organizational problems, or they serendipitously manifest relational behaviours following the evolution of organizational contingences. In the qualitative material that we collected, leaders alternatively use language associated with ‘*managing*’, ‘*manoeuvring*’ and ‘*mastering*’ relationships, and language related to ‘*experiencing*’, ‘*feeling*’ and even ‘*being driven*’ by the happenstance of events. Recent evidence that people can make ‘strategic use’ of networking actions (e.g., Obstfeld, 2005; Soda et al., 2018) should be contextualized in situations of crisis and emergency, which represent jolts shaping both individual behaviours and inter‐individual relationships.

A further element that calls for future research concerns the consequences for organizational functioning of leaders' networking behaviour. What clusters of leaders are more effective in answering the situational demands associated with the COVID‐19 crisis? The premise, which also represents a limitation of this study, is that we do not have enough information in our data that can give answer to this question. This is partly related to our research protocol, which did not emphasize outcomes, and to the emergency context itself, which made the analysis of leader's effectiveness difficult and even ambiguous. What we observed is that, at the macro‐organizational level, the micro‐networking behaviours of individual leaders implied different network consequences for different behavioural clusters. The reshuffle of ties associated with Churners' structural approach to networking led to tie‐level changes that undoubtedly represent opportunities for overall change in network composition (e.g., Chen et al., 2022). The investment in leader‐follower relationships associated with Sense‐makers behaviour, instead, triggered closure dynamics, in which network members consolidated and strengthened existing relationships, making such ties more effective for organizational coordination (e.g., Tasselli, 2015). It is unclear the organizational‐level consequence of Divergents' behaviours: creative abrasion can generate the premise for idea recombination and innovation (e.g., Perry‐Smith and Mannucci, 2017), although interpersonal tension and conflict can be detrimental for organizational cohesion and decision making (e.g., Krackhardt, 1999). More research is needed to give empirical evidence to these insights.

Future work on leaders' agency and organizational effectiveness can also benefit from the preliminary considerations emerging from our data on temporality, which we have already discussed in the findings. For a number of leaders, the upsurge of the emergency made them (relatively) free from formalized and routinized structures, allowing a more unconstrained behavioural expression. But, when the emergency partially faded following the vaccination campaign (phase 3, in June – July 2021), the strength of their behavioural propensities also partially faded in our data. Leaders experienced struggles going back to previous routines and structural arrangements, revealing that the experience of the crisis helped empower the emergence and expression of their authentic relational selves, an expression then again constrained by the resurgence of structure and bureaucracy. Starting from Marxist and Weberian views to recent developments in the field of agency, much has been said by sociological research on the dualism involving (social) structure and (individual) autonomy (see Emirbayer and Mische, 1998). Our study adds to such longstanding debate evidence that this dualism can be part of a continuous process in which behavioural expression needs an appropriate context to be expressed. In this sense, and quite paradoxically, advantageous (but formalized) structural positions could even be detrimental for individual agency. Networks might indeed represent super‐structural dimensions embedding – through group‐level norms and pressures – individual behaviour even when they provide positional advantage to the individual.

### Practical Implications

What can organizations do to empower their leaders, acknowledging and taking into account differences in their networking behaviours? The dilemma we currently face is that organizations often try to boost their social capital intervening on the enablement and development of visible and often formalized interactions between leaders and followers. Examples include internal organizational turnover practices in which formal leaders, for a certain (typically limited) period of time, are involved in blue‐collar jobs (see Amazon); or they attend company events, such as strategy days or retreats, in which leaders and followers have the opportunity to discuss together organizational strategies and to socialize through team‐building and recreational activities. But we know that the very underpinnings of networking behaviour, as we have shown, tend to be engendered through informal, interpersonal and often serendipitous and context‐dependent patterns that influence interpersonal ties, rather than via organizational‐level network event and structures. The problem is even more compelling in a context of crisis, in which leaders face crosscutting situational pressures that can be hard to resolve, considering the impossibility to plan and schedule activities.

What can companies do to reduce the degree of relational information asymmetry associated with organizational crises? First, they can train leaders to face the unexpected. At the beginning of the viral outbreak in Italy, an anesthesiologist had to go beyond the codified guidelines to detect COVID‐19 in the first known Italian patient. As she revealed later in a newspaper interview, ‘I thought that I had to search for something impossible’ (La Repubblica, 2020). Aircraft pilots are trained with virtual reality to face unexpected and even ‘impossible’ situations of crisis and emergency that can boost their reactions to adverse events and nurture their leadership skills. Similar training practices apply to a range of professions, from medical doctors and nurses to military personnel. However, in organizational theory and practice, only limited attention is given to the management of crisis. Can organizations develop emergency training and even simulations to train leaders to boost their relational behaviours in a time of crisis?

Second, organizations can acknowledge that leaders present inherent differences in their relational behaviours and that such differences, as we have shown, become more salient in relatively unconstrained emergency contexts, in which usual rules and routines temporarily vanish. Concretely, companies can proceed in two opposite still complementary directions to reduce the possible distortive effects of behavioural differences on the management of jolts and crises. On the one hand, they can reduce leaders' behavioural uncertainties by investing in the development of guidelines and protocols that leaders can follow when crisis arises. For example, European health‐care systems are currently working on AI‐based tools that can detect, from a set of indicators, the emergence of viral outbreaks and thus activate emergency systems that can guide leaders' behaviour. On the other hand, companies can teach leaders to face uncertainty in their daily job, thus developing their ability to think out of the box. This is one of the goals of internal projects, such as the celebrated Google 20 per cent rule, in which employees are free to assign part of their work time to individual projects that can boost creativity. The development of flexible and emergent skills, in turn, can facilitate informal reactions to situations of crisis.

### Limitations and Conclusion

This study presents several limitations. Despite the relevance of the emergency for our theorizing on leaders' behaviours, we recognize our setting as a quite extreme type of context, given the emphasis on frontline leadership work in one of the world's epicentres during the pandemic. Future work could broaden the analysis to leadership networking in contexts in which organizational crisis calls for the leaders' ability to stabilize the functioning of work‐related interactions. Second, we conducted our analysis from a limited sample of interviews; the medium sample size depended both on the uniqueness of the research context, and on the aim to reach theoretical saturation by interviewing key informants in the crucial phases of the crisis (see Glaser and Strauss, 1967). An important issue concerns the transferability of our research in relation to other contexts of disruption beyond COVID‐19. We believe that the evidence emerging from this case has wider resonance to different types of settings. Behind the leaders' reactions to this emergency, our findings can help illuminate the understanding of relatively homogeneous patterns of relational dynamics that characterize changes in leaders' workplace actions, featuring attention to the micro‐dimensions of ties and behaviours. Third, our main analysis involves data gathered interviewing only leaders. Interviews with a limited number of followers were conducted in a follow‐up phase and used exclusively to run member‐check on the evidence emerging from the main analysis. Considering that followers participate in and contribute to shape and define leaders' actions, more work in needed to investigate relational patterns in contexts in which both leaders and followers foster organization effectiveness.

In conclusion, our exploratory study unveils the nuanced link between individual behaviour and relational patterning in the leadership domain, which becomes particularly salient when leaders and organizations are exposed to emergency pressures. As shown by our research, studying leaders' networking behaviours in a time of disruption implies understanding and locating the micro‐foundational nexus of leaders' action.

## NOTES

[1] To ground the debate in its philosophical development, this dualism was already at the center of Karl Marx''s view of modern leaders as *karakter masken*, i.e. decision makers so compressed by social pressures and normative structures to lose their identities and behaviouralbehavioral autonomies (cf. Granovetter, 1988). A similar view is implicit in the Heidegger''s idea of *Gestell*, i.e. what lies behind or beneath modern social structure. For Heidegger (1927/2010), social structures, including technology, are not simply means to an end, but rather become a mode of human existence, compressing individual behaviourbehavior and her/his ability to lead and exert decision‐making.

[2] Clearly, given its cross‐sectional nature at each point of data collection, the variable ‘Ego network size’ is insufficient to pinpoint network churning in terms of tie creation and tie termination (i.e., turnover of relationships). It merely gauges the overall number of one''s network contacts at a given time point.

[3] The ego‐network captures the leader''s ‘regular contacts’ focused on ‘advice and knowledge transfer, aimed at decision making.’ The variable ‘Ego‐network size T1’ refers to the number of contacts reported in the ego‐network survey completed by the leader during the first phase of data collection, and so on for T2 and T3. During the first phase of data collection, we also asked the leaders to recall the ego‐networks they had before the start of the pandemic. This information, when provided by the leaders, was included as ‘Ego‐network size T0’. The same procedure of data gathering led to the variables ‘Ego‐network tie strength,’ which also include four temporal specifications between T0 and T4. For more details, see Appendix 6.

[4] As illustrated by a recent conceptual piece on network theory, for structural researchers ‘a rock dropped from the same place in the same way has the same outcomes regardless of whether it was dropped on purpose or by accident’ (Borgatti and Halgin, 2011, p. 1178).

[5] For example, a follower of a churning leader (respondent 6) described his behaviourbehavior as ‘He [the leader] always tries to make new connections. He is always involved with others. He spends so much time making new acquaintances.’ Similarly, a follower of a diverging leader (respondent 19) reports: ‘Marc [fictional name] is a person who is totally not afraid of conflict. I would not say he looks actively for conflict, but he never avoids it. Whenever he has to stand for his ideas, he does so, irrespective of whether he has to argue with others or not’.

## Appendix 1 Detailed Information on Respondents and Interviews

**Table:**

Respondent role (formal/informal leader)	F/M	City's size and area	Role in the management of COVID‐19
1. Member of the City Cabinet (formal leader)	M	< 50.000 residents, Milano area	Co‐responsible for the local task force coordination, with focus on service provision for city welfare and social care.
2. Manager of a non‐profit organization providing services to the population (formal leader)	F	< 50.000 residents, Milano area	Responsible for continuing to provide services in a period of disruption.
3. Manager (formal leader)	M	> 100.000 residents, Milano area	Responsible for the coordination between social services and (online) education services.
4. City manager (formal leader)	M	< 15.000 residents, Brescia area	Responsible for coordinating the training for public servants on how to provide services to the population.
5. Member of the City Cabinet (formal leader)	M	< 50.000 residents, Milano area	Co‐responsible for the local task force coordination, with focus on logistics and mobility.
6. Deputy Mayor (formal leader)	M	< 50.000 residents, Milano area	Co‐responsible for the local task force coordination, with focus on the provision of institutional and welfare services.
7. Mayor (formal leader)	M	< 50.000 residents, Milano area	Responsible for the overall running of the municipality, with mandate on task‐force coordination.
8. Entrepreneur (formal leader)	M	> 100.000 residents, Milano area	Responsible for producing and delivering protection clothing.
9. Manager of a non‐profit organization providing services to the youth (formal leader)	F	< 50.000 residents, Milano area	Responsible for continuing to provide services during lockdown and monitor the wellbeing of the youth.
10. Municipality CEO (formal leader)	M	< 100.000 residents, Bergamo area	Responsible for the overall running of the municipality, with mandate on emergency welfare.
11. Deputy Mayor (formal leader)	F	< 100.000 residents, Brescia area	Responsible for the local task force coordination, with mandate on safety and city welfare.
12. Member of the City Cabinet (formal leader)	F	< 50.000 residents, Milano area	Co‐responsible for the local task force coordination, with focus on welfare.
13. Mayor (formal leader)	M	< 50.000 residents, Milano area	Responsible for the overall running of the municipality, with specific mandate on task‐force coordination.
14. Entrepreneur (formal leader)	M	< 50.000 residents, Milano area	Food cooperative president with responsibility for food provision within the city.
15. Vice‐director of media company (formal leader)	M	< 100.000 residents, Milano area	Responsible for digital media communication and information during the crisis.
16. Parish and head of a local centre for the youth (formal leader)	M	< 50.000 residents, Milano area	Responsible for the spiritual care of the community and for the management of a local centre for the youth.
17. Mayor & Vice‐president representative of the Health Territorial Organization (formal leader)	M	< 15.000 residents, Brescia area	Responsible for the overall running of the municipality & for the coordinating the health services institutions in the territorial area.
18. Deputy head of safety and order municipal services (formal leader)	M	< 15.000 residents, Milano area	Responsible for the safety and order of the city during the crisis.
19. Member of the City Council (formal leader)	M	< 100.000 residents, Milano area	Responsible for co‐coordinating the overall municipality response in the management of the crisis.
20. Member of the City Council (formal leader)	M	> 100.000 residents, Milano area	Responsible for co‐coordinating the overall municipality response in the management of the crisis.
21. Manager of a non‐profit organization (formal leader)	F	< 50.000 residents, Milano area	Manager of a local organization providing welfare related services to the population.
22. Municipality Manager (formal leader)	F	< 50.000 residents, Bergamo area	Responsible for providing services within the public works/urban planning sector.
23. Co‐coordinator of commuters'’ society (formal leader)	F	> 100.000 residents, Bergamo area	The society represents commuters' rights and promotes their safety in a dialogue with local and regional institutions during the crisis.
24. Municipality employee (informal leader)	M	< 15.000 residents, Milano area	Municipality employee, recognized as an informal leader, with decades of experience in the provision of social services
25. Inter‐municipality coordinator of logistics (formal leader)	M	< 50.000 residents, Bergamo area	Province‐level representative of municipalities with responsibility for coordination of logistics.
26. Municipality board member (formal leader)	F	< 15.000 residents, Monza‐Brianza area	Municipality board member with responsibility for social services to the population.
27. Informal coordinator of a non‐profit association (informal leader)	F	< 15.000 residents, Monza‐Brianza area	Informal coordinator of an association providing support to elderly people admitted to retirement homes.
28. Informal coordinator of pastoral services (informal leader)	M	< 15.000 residents, Milano area	Informal coordinator of spiritual and pastoral services to the youth
29. Municipality manager (formal leader)	M	< 15.000 residents, Monza‐Brianza area	Municipality manager with responsibility for services related to emergency management
30. Responsible of a non‐profit association (formal leader)	M	< 100.000 residents, Milano area	Responsible of a non‐profit association providing care to homeless people
31. Public manager (formal leader)	M	< 15.000 residents, Milano area	Public manager coordinating the local unit for mobility and transportation
32. Informal coordinator of a non‐profit association (informal leader)	F	< 15.000 residents, Bergamo area	Informal coordinator of an association of parents and relatives of people with disabilities
33. Entrepreneur (formal leader)	M	< 50.000 residents, Monza‐Brianza area	Entrepreneur in the social care sector
34. Coordinator of a non‐profit association (informal leader)	F	< 50.000 residents, Brescia area	Informal coordinator of a non‐profit association of providers of social services
35. Leader of a sport team (informal leader)	M	< 15.000 residents, Milano area	Captain of a rugby team which is part of a multi‐sports club directly involved in local welfare
36. Municipality mid‐manager (formal leader)	M	< 50.000 residents, Milano area	Mid‐manager formally responsible for the emergency unit of a group of municipalities
37. Entrepreneur (formal leader)	F	< 15.000 residents, Milano area	Entrepreneur in the catering industry, with B2B service provision during the crisis
38. Manager of a public‐private company (formal leader)	F	< 15.000 residents, Monza‐Brianza area	Manager of a hybrid public‐private company for welfare services provision to the population
39. Municipality employee (informal leader)	M	< 50.000 residents, Milano area	Employee of the municipality public library, with decades of experience and recognized informal coordination of local services to the population
40. Municipality board member (formal leader)	F	< 15.000 residents, Monza‐Brianza area	Municipality board member with responsibility for the overall provision of services to the person
41. Entrepreneur (formal leader)	M	< 50.000 residents, Monza‐Brianza area	Entrepreneur in the field of local mobility and transportation
42. Informal coordinator of a non‐profit association (informal leader)	F	< 15.000 residents, Milano area	Informal coordinator of an association of researchers and teachers that offer free education services to the population

*Notes*: To ease interpretation, we classified cities' size according to the following criteria: < 15.000 residents, < 50.000 residents, < 100.000 residents, > 100.000 residents.

## Appendix 2 Selected Questions from Evolving Semi‐structured Interview Protocol Used in Phase 1 of the Data Collection (21 February – 21 April 2020)

With sub‐phase 1 of the research, we identify the period from 21 February 2020 to 14 March 2020 (‘The outbreak’); with sub‐phase 2, the period from 15 March 2020 to 5 April 2020 (‘The lockdown and the peak’); and with sub‐phase 3, the period from 6 April 2020 to 21 April 2020 (‘Starting to plan the next phase’).

**[Part A]. Introduction and overview of job and leadership role**. (Throughout sub‐phase 1 and sub‐phase 3).

Before the official start of the interview, in addition to the procedure related to the explicit request of informed consent, for each respondent we read a statement. ‘All your answers to the questions of this interview will be held strictly anonymous, such that neither you, nor the subjects you refer to, or your organization will be identified as taking part to this research’. We also asked the respondent's permission to record the interview. If denied, we asked permission to take extensive notes, and to validate these notes, including verbatim expressions, with the respondents after the interview for their approval. We also asked permission to use some of the quotes from the interview, respecting the anonymity of the respondent, for research purposes only related to this study.

**Introduction**. Thank you for your availability for this interview. We will ask you questions concerning your job, your role and your interactions with employees, citizens and other actors in the community. We will focus specifically on change in personal, professionals and relational aspects following the beginning of the recent COVID‐19 health emergency. We would ask you to be as open as you can in your answers to these questions. First, tell us a little about yourself and your job.

1. What is your current position? What are the main tasks and responsibilities associated with this position? How many people do you directly coordinate/supervise, and, more specifically, for what kinds of tasks?
2. How long have you been in this position?
3. Tell me a little more about your role. What did you do in a typical day before the emergency? What kinds to activities did you usually perform?
4. How do you manage your role of coordinator/supervisor? What are the main positive elements of your role? What are the main challenges? Overall, are you satisfied with your role? What would you eventually change, or improve?

**[Part B]. Effect of the COVID‐19 emergency on job and role.** (Questions 1 to 2 throughout sub‐phase 1 and sub‐phase 3; question 3 only in sub‐phase 1; question 4 added in sub‐phase 2 and sub‐phase 3).

1. Can you tell us more about the day in which the COVID‐19 emergency started in your organization/local community? What were you doing? What have been your immediate thoughts and reactions? What did you tell your employees, supervisees or collaborators? What did they tell you?
2. How did the emergency change your personal work routine? Can you describe the main changes that occurred in an average workday? What are your impressions, personal experiences or opinions about these changes?
3. What means leading your organization/local community in time of COVID‐19? How does this situation change your conception of your job and your role? How does it change your responsibilities towards your employees and collaborators, and towards your community? How do you experience, personally and subjectively, these changes?
4. Think about the effects of the lockdown on your role. How did it change your leadership in the organization and in the local community? Can you give us examples? How did you experience, personally and subjectively, the lockdown?

**[Part C]. Effects of the COVID‐19 emergency on institutional, professional and interpersonal interactions and collaborations.** (Questions 1 to 4 throughout sub‐phase 1 and sub‐phase 3; In sub‐phase 2 and 3, we included in each question explicit mention of the lockdown. Questions 5 and 6 added in sub‐phase 2 and sub‐phase 3. The expression ‘in the last few weeks, after the start of the emergency’ was used in sub‐phase 1; in sub‐phase 2 and 3 of the research, we used only the expression ‘since the beginning of the emergency’).

1. Think about your meetings with other relevant stakeholders in the organization/local community before and after the start of the emergency. What are the main changes – if any – that you evidence? How did your interaction with these stakeholders change? Can you report examples – if any – of collaboration? Can you report examples – if any – of tension or conflict?
2. Think about your professional interaction with your employees, supervisees or collaborators. Did you notice any change in the last few weeks, after the start of the emergency? Do you think that the way you interact with them is effective in this situation?
3. Think about your personal interaction with your employees, supervisees or collaborators. What personal changes (from your side or from their side) did you notice in the last few weeks, after the start of the emergency?
4. Now, think about your relationships with stakeholders or citizens in the community. What changes – if any – did you experience? Did you have to solve any problem that required your interaction with the community?
5. How did the lack of physical contacts with others shape your opportunities of interactions with employees, supervisees and collaborators, and, more in general, with the community?

[**Part D]. Effects of the COVID‐19 emergency on the leader**'**s personal approach to the role and to the interactions, and future prospects.** (Questions 1 and 2 throughout sub‐phase 1 and sub‐phase 3; question 3 was included only in sub‐phase 1, and substituted with question 4 in sub‐phase 2 and 3. Question 5 was included only in sub‐phase 3. The expression ‘in the last few weeks’ was used in sub‐phase 1; in sub‐phase 2 and 3 of the research, we used the expression ‘since the beginning of the emergency’).

1. During these weeks, did you change your approach to your own job and role? Do you experience, from a subjective perspective, what you are doing in a different way than before? What are your feelings about this change?
2. During these weeks, did you notice – if any – relevant changes in your personal approach to your employees, supervisees or collaborators? Are you dealing with them in different ways than before? What are your opinions, personal experiences and impressions about your interactions in this evolving situation?
3. How do you expect your job and your role to change in the future, if the emergency continues?
4. Considering the current uncertainty about the evolution of the situation, what can you say about the expectations that you have about your job and your role in the future?
5. In the last week(s), the statistics report a decline in the number of new contagions. Did this evidence change in any way your role and your interactions?

## Appendix 3 Selected Questions from Evolving Semi‐structured Interview Protocol Used in Phase 2 of the Data Collection (15 November – 21 December 2020)

With sub‐phase 1 of the research, we identify the period from 15 November 2020 to 3 December 2020 (‘Red zone, or second total lockdown’); with sub‐phase 2, the period from December 4 2020 to 21 December 2020 (‘Orange and yellow zone, or after total lockdown’).

**[Part A]. Introduction.** (Throughout sub‐phase 1 and sub‐phase 2).

Before the official start of the interview, in addition to the procedure related to the explicit request of informed consent, for each respondent we read a statement. ‘All your answers to the questions of this interview will be held strictly anonymous, such that neither you, nor the subjects you refer to, or your organization will be identified as taking part to this research’. We also asked the respondent's permission to record the interview. If denied, we asked permission to take extensive notes, and to validate these notes, including verbatim expressions, with the respondents after the interview for their approval. We also asked permission to use some of the quotes from the interview, respecting the anonymity of the respondent, for research purposes only related to this study.

**Introduction.** Thank you for your availability for this interview, which follows the interview that we conducted in early 2020. At that time, we asked you questions concerning your job, your role and your interactions with employees, citizens and other actors in the community, focusing specifically on change in personal, professionals and relational aspects following the beginning of the recent COVID‐19 health emergency. In this second interview, we will ask you again about the management of the COVID‐19 crisis, focusing on what you and your organization have learned from the first wave, on how it can help face this second wave. More specifically, on how you built or changed your personal relationships with followers and stakeholders, and on the ways you reacted to the crisis. As we did in the first interview, we would ask again you to be as open as you can in your answers to these questions. First, tell us a little about yourself and your job.

1. Do you still occupy the same position you occupied in early 2020? Do you still have the same role and responsibilities? Do you coordinate the same number of people? If anything changed, can you detail the change? Was the (eventual) change related to the COVID‐19 crisis?

**[Part B]. Effect of the second wave on the person, job and role.**

1. Can you tell us more about how the second wave affected your organization/ local community? What were the main differences with the first wave of the emergency? What did your organization/local community learned from the first wave that was relevant in this second wave?
2. Are you experiencing now the same changes in your work role/routine that you experienced during the first phase of the emergency? If not, what is different now from then? How can you explain this difference?
3. Relative to the first wave, are you experiencing now different ways in which you lead your organization/local community? If so, how can you explain these changes?
4. What is your personal reaction to this second wave? Did you expect it, or was it unexpected? How did it affect the way you subjectively approach your role and your profession? What are your feelings in this second wave the crisis, and how are they eventually different from the first wave?

**[Part C]. Effects of the second wave on the personal reaction to the crisis.**

1. Compared to the first wave, what have been (if any) the main differences in the personal reaction to the crisis, in terms, for example, of sentiments, beliefs, feelings? Can you mention real‐life examples?
2. In this second wave, did anything that you have learned during the first wave help you manage your personal reactions to the crisis? If so, what did you learn, and how did it help? Please provide examples.
3. Did anything that others learn from the first wave – in your team and beyond it – help you manage your personal approach to the crisis? If so, please provide examples.

**[Part D]. Effects of the second wave on interpersonal and social interactions and collaborations.**

1. How did your professional interactions with others (stakeholders, followers, other actors) change in this second wave of the pandemics, compared both to the first wave, and to the summer period? Can you mention relevant examples? Please emphasize, if possible, both collaboration and conflict.
2. How did your personal interactions change? Did you experience any difference in the personal and subjective way you interact with others in this second phase, compared to the previous one?
3. What initiatives did you establish and follow to create/maintain personal interactions with others during this second lockdown? Are you experiencing any difference compared to the previous wave? If so, please mention relevant examples.

[**Part E]. Effects of the second wave on the leader**'**s personal approach to herself and to others, and next steps.**

1. What is your subjective reaction to this second wave, in terms of behaviours, feelings, beliefs? Are you experiencing, at the personal level, any difference relative to the first phase?
2. Did you notice – if any – relevant changes in your personal approach to your employees, supervisees or collaborators, relative both to the first wave and to summer?
3. What do you expect for the near future? Overall, how will this pandemic affect your leadership role and future trajectory, when it will be over?

## Appendix 4 Selected Questions from Evolving Semi‐structured Interview Protocol Used in Phase 3 of the Data Collection (9 June – 27 July 2021)

With sub‐phase 1 of the research, we identify the period from 9 June to 30 June 2021 (‘Before the application of the EU Digital COVID Certificate’); with sub‐phase 2, the period from July 1 and July 27 2021 (‘After the application of the EU Digital COVID Certificate’).

**[Part A]. Introduction.** (Throughout sub‐phase 1 and sub‐phase 2).

Before the official start of the interview, in addition to the procedure related to the explicit request of informed consent, for each respondent we read a statement. ‘All your answers to the questions of this interview will be held strictly anonymous, such that neither you, nor the subjects you refer to, or your organization will be identified as taking part to this research’. We also asked the respondent's permission to record the interview. If denied, we asked permission to take extensive notes, and to validate these notes, including verbatim expressions, with the respondents after the interview for their approval. We also asked permission to use some of the quotes from the interview, respecting the anonymity of the respondent, for research purposes only related to this study.

**Introduction.** Thank you for your availability for this interview, which follows the interviews that we conducted in early and in late 2020. At that time, we asked you questions concerning your job, your role and your interactions with employees, citizens and other actors in the community, focusing specifically on change in personal, professionals and relational aspects following the two waves of the COVID‐19 health emergency in 2020. In this third interview, we will ask you again about the management of the COVID‐19 crisis, but, considering the moment of relative non‐emergency that we are experiencing, we will focus more on what you have learned from the crisis in relation to your job, to your profession, to your leadership role and to the way you interact with others.

1. Do you still occupy the same position you occupied in early and late 2020? Do you still have the same role and responsibilities? Do you coordinate the same number of people? If anything changed, can you detail the change? Was the (eventual) change related to the COVID‐19 crisis or to the aftermath of it?

**[Part B]. What the leader has learned from the crisis**.

1. In the previous interviews, you told us about how the crisis affected your work and your interactions with others? Now, if you look back at what happened in this last 18 months, what have you learned, which you did not know before, about your job, your tasks, your activities?
2. If you look back at February 2020, before the emergency, and if you look at your work now, what has changed? Can you focus specifically on three main points concerning your job, your tasks and your activities?
3. If you look back at February 2020, before the emergency, and if you look at your work now, what have you learned from it as a leader? For example, what would you do differently in your leadership role? Again, if you can, please focus on three key points.
4. Focus now on the ways you lead others (employees, stakeholders, etc.) and on the ways you lead your organization/local community? What has changed from early 2020 to today?
5. What have you learned as a person? How did this experience and your leadership work during the emergency change you as a person, and how did it change your interactions with others?

**[Part C]. Interaction with others and relational approaches to the role.**

1. What have you learned as a person? How did this experience and your leadership work during the emergency change you as a person, and how did it change your interactions with others?
2. What did you learn about the ways to manage relationships with others? What would you do differently today? Can you give us concrete examples?
3. Think about your interactions with others in these 18 months. How did the way you manage your interactions with others help you to be an effective leader during the emergency? Can you give us concrete examples?
4. Related to the question above, what did not work so well in the way you interacted with others? Can you give us a few examples? You can focus on organizational or relational

[**Part D]. Next steps.**

1. What is your subjective reaction to overall emergency, in terms of behaviours, feelings, beliefs? Are you experiencing, at the personal level, any change that you think will endure also when the crisis will be finally finished?
2. Did you notice – if any – relevant changes in your personal approach to your employees, supervisees or collaborators, relative to before the crisis and to the peak of the emergency?
3. What do you expect for the future? Overall, how will this pandemic affect your leadership role and future trajectory? How did it shape the way you behave as a leader? Can you give us a few examples or learnings?

## Appendix 5 Selected Questions from the Semi‐Structured Protocol Used to Interview a Leader's Followers

*Note*: the names of the respondents were provided by the leaders themselves, who also authorized us to interview these informants.

**[Part A]. Introduction.**

Before the official start of the interview, in addition to the procedure related to the explicit request of informed consent, for each respondent we read a statement. ‘All your answers to the questions of this interview will be held strictly anonymous, such that neither you, nor the subjects you refer to, or your organization will be identified as taking part to this research’. We also asked the respondent's permission to record the interview. If denied, we asked permission to take extensive notes, and to validate these notes, including verbatim expressions, with the respondents after the interview for their approval. We also asked permission to use some of the quotes from the interview, respecting the anonymity of the respondent, for research purposes only related to this study.

**Introduction.** Thank you for your availability for this interview. We will ask you questions relative to the relational behaviour of [name of the leader] in the different phases of the management of the COVID‐19 emergency.

1. What is your role/position? What was your role during the COVID‐19 crisis? What was your (workflow/formal/prescribed) interaction with [name of the leader]?

**[Part B]. Leader**'**s behaviour during the crisis.**

1. If you look back at the entire timeline of the COVID‐19 crisis, since the beginning of the emergency till now, how would you describe the actions and behaviours of [name of the leader] in the management of COVID‐19 within/beyond your organization? Please formulate examples.
2. Focus now on the ways [name of the leader] contributed to lead your organization/local community during this timeline. What has changed from early 2020 to today? How would you describe her/his behaviour? Again, if you can please mention real‐life examples.

**[Part C]. Leader**'**s interactions with the respondent and with others.**

1. During the management of COVID‐19, how did [name of the leader] interact with you? Based on your knowledge, how did she/he manage relationships with others? Can you give us concrete examples?
2. Think about [name of the leader]'s interactions with others in the analysed timeline. What did the way [name of the leader] managed interactions with others tell us about her/his leadership abilities?
3. What can we learn from [name of the leader]'s style in interacting with others? Please focus on key points.

## Appendix 6 Intensity Scales for the Six Networking Actions: Representative Data

**Table:**

Networking Domains and Actions	Representative data for Scale Endpoints
*Networking domain: Dynamics in ego‐network structure*	
1. Network generation	*Quote representative of value ‘1’* (*very low level of network generation*) ‘I do not have any particular interest in making new connections at this point in time. I want to leverage the connections I already have because they can be particularly useful. Now, creating new connections could be confusing rather than good’. (Respondent 21) *Quote representative of value ‘5’ (very high level of network generation)* ‘It's all about making your network larger. New devices? You must create the tie with those who can help you with that. Receiving advice on how to activate the procedure [name of the procedure]? You must form a tie with those actors who know how to activate the procedure. And so on. It's all about being able to go beyond the ties you have and form new ties. It is a continuous effort in having new ties’. (Respondent 8)
2. Network termination	*Quote representative of value ‘1’* (*very low level of network termination*) ‘Especially when we had to face that huge shock, we had to be close to each other and united. I was hearing the ringing of sirens at day and night. How could I drop a connection with somebody in my community? It was a moment of union’. (Respondent 4) *Quote representative of value ‘5’* (*very high level of network termination*) ‘You know, this was also the chance to see who did not provide value. In normal life, this does not happen often. Here, almost every day. There was no filter. I would not call it instrumentalism, but necessity to cut connections due to the emergency. The more you cut, the more you keep the ones that provide value’. (Respondent 38)
*Networking domain: Dynamics in network utilization*	
3. Network conflict.	*Quote representative of value ‘1’* (*very low level of network conflict*) ‘Come on, how can people be confrontational when there is this kind of unimaginable situation? Unity, cohesion, even homogeneity of thoughts. Certainty, not useless individualistic debates’. (Respondent 6) *Quote representative of value ‘5’* (*very high level of network conflict*) ‘I remember we had this open argument, for almost half an hour, with very frank positions and even with a strong confrontational spirit. It was the key to be open to each other. In that phase, it happened quite often, and I think was super helpful to find solutions to open issues’. (Respondent 19)
4. Network deepening	*Quote representative of value ‘1’* (*very low level of network deepening*) ‘There was no time to spend energy in understanding their thoughts. It was the moment of action, not the one of getting to know what they had in mind. In the end, at that time we were so stressed that we could not even listen to ourselves, so there was surely no time to listen to others' thoughts’. (Respondent 2) *Quote representative of value ‘5’* (*very high level of network deepening*) ‘When we say, “we know somebody”, what do we mean? This crisis gave the possibility to invest time, energy, emotional closeness towards understanding each other better. We spent so much time together, in this difficult situation, which became almost natural to invest more energy in the reciprocal effort to know each other better, to make the effort to see the world from the perspective of the other person, without prejudice, without individualism’. (Respondent 28)
*Networking domain: Dynamics in network interpretation*	
5. Network teleology	*Quote representative of value ‘1’* (*very low level of network teleology*) ‘No time to understand the “big why”. It applied to each of us and to all of us collectively. Only time for solutions. It did not matter what was the underlying goal of each of us individually, because the pandemic cancelled any reflection of this kind’. (Respondent 37) *Quote representative of value ‘5’* (*very high level of network teleology*) ‘For the first time, I looked at her [a colleague], I looked at him [another colleague] and tried to understand the big purpose of our “being together”. Maybe there were mysterious reasons explaining why we were there together, why we had to face all of this. I could not answer this question, but such question changed irremediably my way to look at them. Everything became more intimate and personal’. (Respondent 33)
6. Network re‐construal	*Quote representative of value ‘1’* (*very low level of network re‐construal*) ‘Come on, John [fictional name. The respondent recalls a conversation with a co‐worker]. Do you believe there is time for understanding what did not work between us, and understand how our interpersonal and professional relationship can work? There is no time for it, not at all. There is just time to answer emails and make phone calls to try to get out of this terrible moment’. (Respondent 34) *Quote representative of value ‘5’* (*very high level of network re‐construal*) ‘No, it's not about giving a second change. It's about building these personal interactions on a more solid foundation. Changing the nature of these relationships can be good, but implies the effort to see what worked and what did not work, and the will to shift their meaning and their focus. It is something we do often with our friends, barely with colleagues, but now we have the chance to do it’. (Respondent 40)

## Appendix 7 Methodology of the Ego‐Network Data Collection

To have a more comprehensive understanding of the leaders' networks, we collected ego‐network data (for the detailed procedure, see Wasserman and Faust, 1994), asking each leader in each of the three phases ‘with whom she/he had regular contacts, including advice and knowledge transfer, aimed at decision making’. During the first phase, we also asked the leaders to recall their networks before the pandemic. Due to our access at that time to leaders only and not to followers, we only collected information on leaders' direct ties (i.e., leader‐to‐alter tie), and not information on ties between alters (i.e., alter‐to‐alter tie). This led us to retain two categories of structural information for each leader, used to create associated variables adopted to compare clusters (for mean values and standard deviations across clusters, see Table V): *ego‐network size*, i.e., the number of ties that leaders list in their ego‐networks (e.g., if a given leader mentions four ties at time 1, the value for that leader of the ego‐network size measure at T1 = 4); and *ego‐network strength*, i.e., the average strength of leaders' ties to followers, as reported by the leader (ranging from 1 to 5, where ‘1’ = limited weekly contact with the follower, and ‘5’ = intense contact with the follower multiple times per working day; e.g., Tortoriello and Krackhardt, 2010) (e.g., if a given leader reports at time 1 two ties, and reports the tie strength with the first contact being ‘4’ and the tie strength with the second contact being ‘5’, the value for that leader of the ego‐network strength measure at T1 = 4.5). For each leader, we assessed measures of ego‐network size and average ego‐network strength at T0, T1, T2 and T3.
